# Quality traits analysis of 153 wheat lines derived from CIMMYT and China

**DOI:** 10.3389/fgene.2023.1198835

**Published:** 2023-08-02

**Authors:** Pengpeng Liu, Zhe Zhang, Yuruo Yin, Shanshan Yan, Yong Ren, Wei Sang, Hongjun Xu, Xinnian Han, Fengjuan Cui, Yingbin Nie, Dezhen Kong, Wei Li, Caixia Lan, Peiyuan Mu

**Affiliations:** ^1^ Institute of Crop Research, Xinjiang Academy of Agri-Reclamation Sciences, Key Lab of Xinjiang Production and Construction Corps for Cereal Quality Research and Genetic Improvement, Shihezi, Xinjiang, China; ^2^ National Key Laboratory of Crop Genetic Improvement, Hubei Hongshan Laboratory, College of Plant Science and Technology, Huazhong Agricultural University, Wuhan, Hubei, China; ^3^ Mianyang Institute of Agricultural Science, Crop Characteristic Resources Creation and Utilization Key Laboratory of Sichuan Province, Mianyang City, Sichuan, China

**Keywords:** wheat, quality property, quality related genes, molecular markers, KASP marker

## Abstract

In order to understand the difference of quality for Chinese and CIMMYT wheat varieties (lines), we selected 153 wheat germplasm from both China and CIMMYT to explore the contribution relationship of different allelic variation combinations to wheat quality through genotyping and phenotyping, including grain hardness, polyphenol oxidase (PPO) activity, lipoxygenase (LOX) activity, yellow pigment (YP) content and protein content. In terms of flour milling quality, Chinese wheat varieties were mainly carrying *Pina-D1a/Pinb-D1b*, accounting for 32.0% of the total tested varieties, while the CIMMYT wheat lines were mainly carrying *Pina-D1b/Pinb-D1a* with 45.8% of the total collection. The distribution frequencies of subunit 1/2* and 5 + 10 were 47.0% and 42.5%, respectively, in CIMMYT varieties, however they were only 31.4% and 13.7% respectively of the Chinese wheat tested varieties. In addition, the proportion of phytoene synthase (PSY) allele, PPO allele and LOX active allele were roughly the same between Chinese and CIMMYT varieties. Based on the present study, we found that *Pina* gene had a greater impact on grain hardness value than *Pinb* gene; The influence of *PPO-A1* gene on polyphenol oxidase activity was more significant than *PPO-D1* gene. The high protein content of varieties mostly containing hardness genes and 1/2*/5 + 10 subunit combinations. Based on the present study, we found that the quality gene distribution of Chinese and CIMMYT varieties was quite different, for instance, the high-quality HMW-GS subunits of Chinese varieties were lower than CIMMYT lines. It will be much useful for Chinese wheat breeders to develop good quality wheat variety by crossing with 3 good strong gluten CIMMYT wheat lines by molecular marker-assisted selection.

## Introduction

Wheat is one of the most important food crops in the world. China is the largest wheat producer and consumer worldwide, accounting for about 1/6 of the global wheat yield production. Although China has high production and inventories of wheat, there is still a large gap of good quality wheat resulting in a huge import (Sun et al., 2017; Wang et al., 2019). With the improvement of people’s living standards, the demand of good quality bread continues to grow, leading to structural imbalances between supply and demand. Therefore, it is necessary to accelerate the improvement of wheat quality through molecular marker-assisted analysis of quality genes and phenotype of quality traits.

Wheat quality was mainly affected by genetics. For example, wheat grain hardness is regulated by a pair of major genes *Ha*, which is controlled by *Pina* and *Pinb* (*Pins*) genes, respectively. The loss of *Pina* protein or the mutation of *Pinb* protein gene can cause the change of wheat from soft to hard (Chen et al., 2010). Grain hardness is one of the key traits that determine wheat milling quality. In addition, it affects wheat flour yield, moisture content and processing quality, etc. (Morris, 2002; Chen et al., 2005; Liu et al., 2015). High molecular weight glutenin subunits (HMW-GS) are one of the measurement standards of processing quality. Its content in grains is low, but it has a significant impact on quality, such as whiteness, wet gluten content, water absorption and stability (Zhang et al., 2019). Wheat HMW-GS is encoded by the *Glu-1* locus on the long arms of the wheat chromosomes 1A, 1B and 1D, which are *Glu-A1*, *Glu-B1* and *Glu-D1*, respectively. Each site produces two subunits, named as x and y subunits (Zhang et al., 2016). The high-quality subunits, namely, Ax1 or Ax2*(1/2*), Dx5+Dy10 (5 + 10) and their combinations have a positive effect on the strength of gluten (Payne, 1987; Cooper et al., 2015; Vancini et al., 2019; Guzmán et al., 2022; Sönmez et al., 2023).

Wheat yellow pigment (YP), polyphenol oxidase (PPO) activity, and grain lipoxygenase (LOX) activity mainly affect the quality of wheat flour. In China, the color of flour is a crucial trait of wheat quality evaluation. Phytoene synthase (Psy) is a key enzyme of yellow pigment in flour, which plays a significant role in yellow pigment in flour. Previous studies showed that one of the major QTL on wheat chromosome 7A showed significant association with yellow pigment content, which can explain 60% of YP phenotypic variation (Kuchel et al., 2006). PPO activity is related to browning and impacts the whiteness of flour. High PPO activity is able to cause browning in flour processing and storage resulting in reduction the color of flour, while low PPO activity can increase white color of flour. The major genes that control the PPO activity of wheat grains were located on chromosomes 2A, 2B, and 2D (Zhang et al., 2006). Furthermore, the genes on chromosomes 1AL and 4BS have a major effect upon wheat LOX activity (Geng et al., 2012). LOX can couple and oxidize the carotenoids in wheat flour, which can make wheat flour white. Notwithstanding, excessive LOX activity will destroy the yellow pigment of wheat, making wheat flour too white and losing many nutrients (Han et al., 2015).

At present, the low-quality wheat inventory is high, while the high-quality wheat gap is large. It has become an important issue to explore the quality differences of core germplasm at home and abroad, accelerate the cultivation of high-quality wheat, and improve the structural supply-demand relationship in China. At the present study, we collected 153 wheat germplasm from Chinese and CIMMYT and genotyped with molecular makers of known genes and phenotyped them with the related traits. The objectives of this study are 1) to analyze the differences of wheat quality between Chinese and CIMMYT wheat germplasm; 2) to identify good quality wheat germplasm for wheat breeder to develop new wheat varieties; 3) to figure out the best quality gene combinations for achieving good quality wheat varieties.

## Results

### Genotypes of wheat quality related genes for a germplasm collection

**FIGURE 1:**
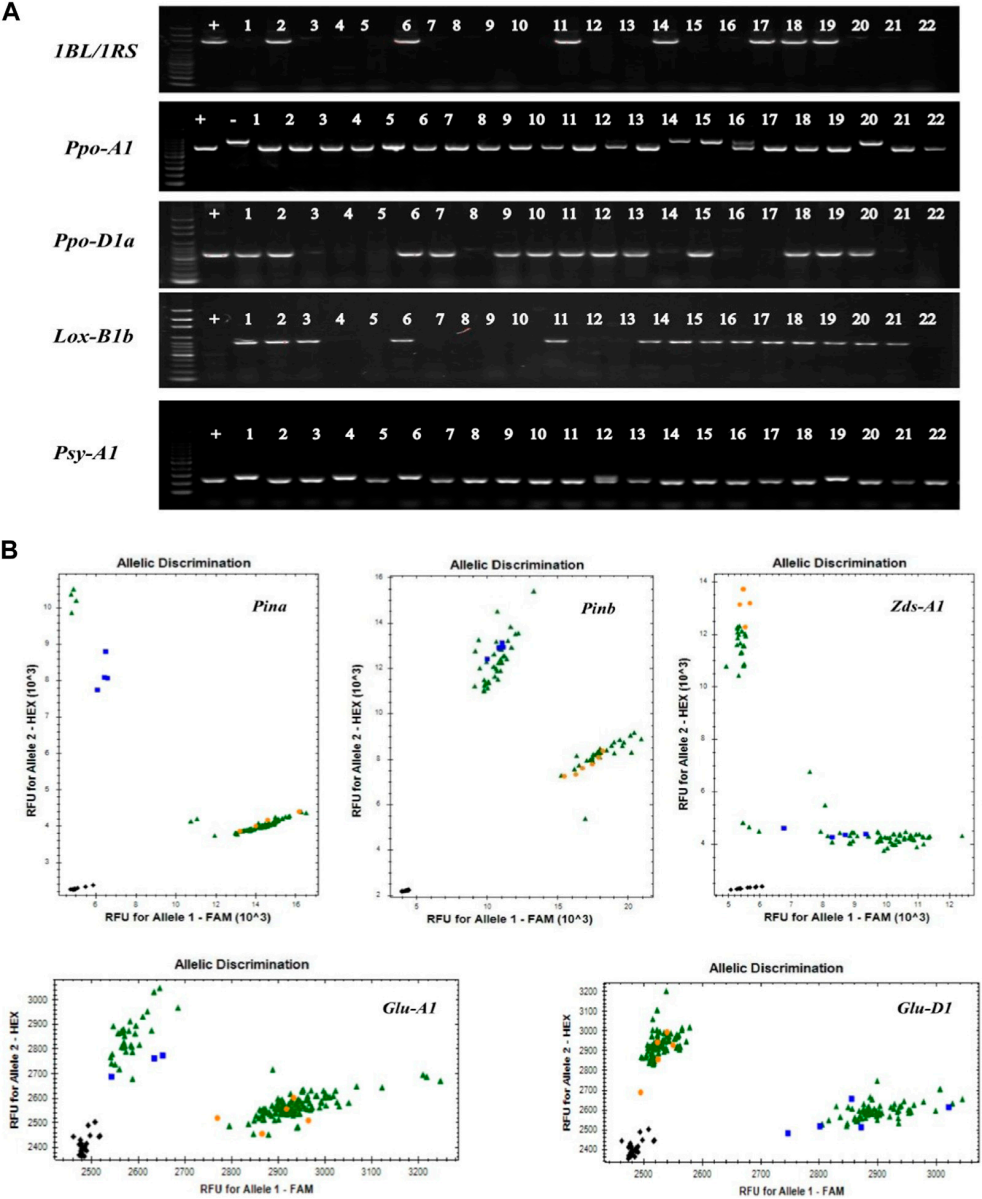
Part of PCR amplification of wheat cultivars. The detection results of partial materials with quality genes display **(A)** Common PCR results. +, Positive control; −, Negative control; 1–22, Test materials; **(B)** Kasp detection results of *Glu-A1* and *Glu-D1*. Black, ddH2O; Blue and Orange, control varieties; Green, test material.

**FIGURE 2:**
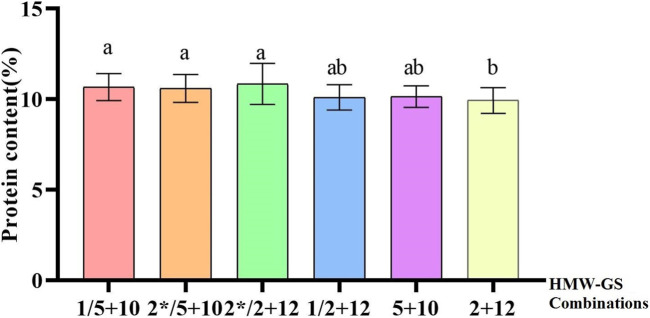
Known quality gene distribution of 153 materials based on the functional molecular markers.

Based on the previous studies, we collected most of the wheat quality related molecular markers and summarized them in the Supplementary Table S1. We used all of these molecular markers to genotype 153 wheat collection from China and CIMMYT (Figure 1; Supplementary Table S2). The result showed that the frequency of 1BL/1RS translocations was 17.6%, while it was 41.8% and 62.1% of alleles *Ppo-A1b* and *Ppo-D1a*, respectively, those were correlated with the lower PPO activity. The frequencies of *Psy-A1b* and *Zds-A1a* were 32.7% and 31.4%, respectively, and they were associated with the lower YP content. The proportions of hardness genes, *Pina-D1b* and *Pinb-D1b*, were 48.4% and 32.7%, respectively, while *LOX-B1b* was accounted for 78.4% that was relevant to the lower LOX activity. The frequencies of 1/2* and 5 + 10 were 78.4% and 56.2%, respectively, association with the HMW-GS subunits (Figure 2).

### Distribution of allelic variants among the collection

In terms of appearance quality, the distribution frequency of Chinese and CIMMYT wheat varieties is roughly the same among different genes or subunit combinations. There were four different types of allelic variants in the two major genes, *Ppo-A1* and *Ppo-D1* that control the activity of PPO. The PPO activity of the combination containing *Ppo-A1a* gene was markedly higher than that of other variant types. The activity of *Ppo-A1b/Ppo-D1a* was significantly lower than that of *Ppo-A1a/Ppo-D1b*, *Ppo-A1a/Ppo-D1a*, *Ppo-A1b/Ppo-D1b*. PPO activity varied greatly among different wheat varieties, and it was ranged from 114.7 to 898.0 and from 90.4 to 756.9 across 2-year data (Table 1). The variation range of yellow pigment content was 0.94–3.47 and 0.93–2.91 over 2 years, while there was no significant difference of YP content trend between different genotypes. The *Psy-A1a/Zds-A1b* gene combination that controls the yellow pigment content was obviously higher than that of *Psy-A1b/Zds-A1b* and *Zds-A1a/Psy-A1b* (Table 2). In addition, the LOX activity was significantly higher for wheat varieties containing *LOX-B1a* gene than that of *LOX-B1b* (Table 3).

**TABLE 1:** Comparison the PPO activities among different genotypes.

Year	Genotype	Domestic varieties number	CIMMYT varieties number	PPO activity of whole wheat flour AU/(min*g)			
				Mean^a^	SD^b^	CV(%)^c^	Range
2020	*Ppo-A1a/Ppo-D1b*	19	16	600.2^A^	108.3	18.0	417.0–867.7
	*Ppo-A1a/Ppo-D1a*	28	26	481.4^B^	79.8	16.6	148.3–898.0
	*Ppo-A1b/Ppo-D1b*	12	11	301.8^C^	55.4	18.4	114.7–364.2
	*Ppo-A1b/Ppo-D1a*	22	19	237.5^D^	68.0	28.7	127.0–671.0
2021	*Ppo-A1a/Ppo-D1b*	19	16	501.2^A^	110.4	22.0	274.8–756.9
	*Ppo-A1a/Ppo-D1a*	28	26	469.3^A^	69.9	14.9	90.4–739.8
	*Ppo-A1b/Ppo-D1b*	12	11	273.7^B^	60.4	22.1	116.3–345.3
	*Ppo-A1b/Ppo-D1a*	22	19	232.7^B^	64.4	27.7	93.1–543.9

^a^ Different letters following the mean indicate significant differences based on a *t*-test (*p* < 0.01).

^b^ SD, standard deviation.

^c^ CV, coefficient of variation in percent.

Ppo-A1b/Ppo-D1a, PPO gene variation site.

**TABLE 2:** Comparison the YP content among different genotypes.

Year	Genotype	Domestic varieties number	CIMMYT varieties number	YP content of flour (mg/kg)			
				Mean^a^	SD^b^	CV(%)^c^	Range
2020	*Psy-A1a/Zds-A1b*	42	33	2.3^A^	0.46	19.5	1.46–3.47
	*Psy-A1a/Zds-A1a*	14	14	2.2^A^	0.49	21.9	1.59–3.46
	*Psy-A1b/Zds-A1b*	30	10	1.9^B^	0.52	28.2	0.98–3.08
	*Zds-A1a/Psy-A1b*	5	15	1.6^C^	0.36	23.1	0.94–2.31
2021	*Psy-A1a/Zds-A1b*	42	33	1.9^A^	0.40	21.1	1.17–2.86
	*Psy-A1a/Zds-A1a*	14	14	1.8^A^	0.39	21.3	1.26–2.91
	*Psy-A1b/Zds-A1b*	30	10	1.4^B^	0.34	23.9	1.12–1.97
	*Zds-A1a/Psy-A1b*	5	15	1.3^B^	0.19	14.8	0.93–1.54

^a^ Different letters following the mean indicate significant differences based on a *t*-test (*p* < 0.01).

^b^ SD, standard deviation.

^c^ CV, coefficient of variation in percent.

Psy-A1a/b and Zds-A1a/b, yellow pigment comment gene variation site.

**TABLE 3:** Comparison the LOX activities among different genotypes.

Year	Genotype	Domestic varieties number	CIMMYT varieties number	LOX activity of whole wheat flour (Nkat*g-1)			
				Mean^a^	SD^b^	CV(%)^c^	Range
2020	*LOX-B1a*	26	7	16.8^A^	4.13	24.6	11.3–27.0
	*LOX-B1b*	55	65	13.9^B^	4.06	29.3	3.3–22.7
2021	*LOX-B1a*	26	7	17.8^A^	3.07	20.7	10.8–25.7
	*LOX-B1b*	55	65	13.4^B^	3.67	22.9	7.4–22.1

^a^ Different letters following the mean indicate significant differences based on a *t*-test (*p* < 0.01).

^b^ SD, standard deviation.

^c^ CV, coefficient of variation in percent.

L0X-B1a/b, LOX gene variation site.

### Identification and origin of good wheat quality germplasm among the collection

**FIGURE 3:**
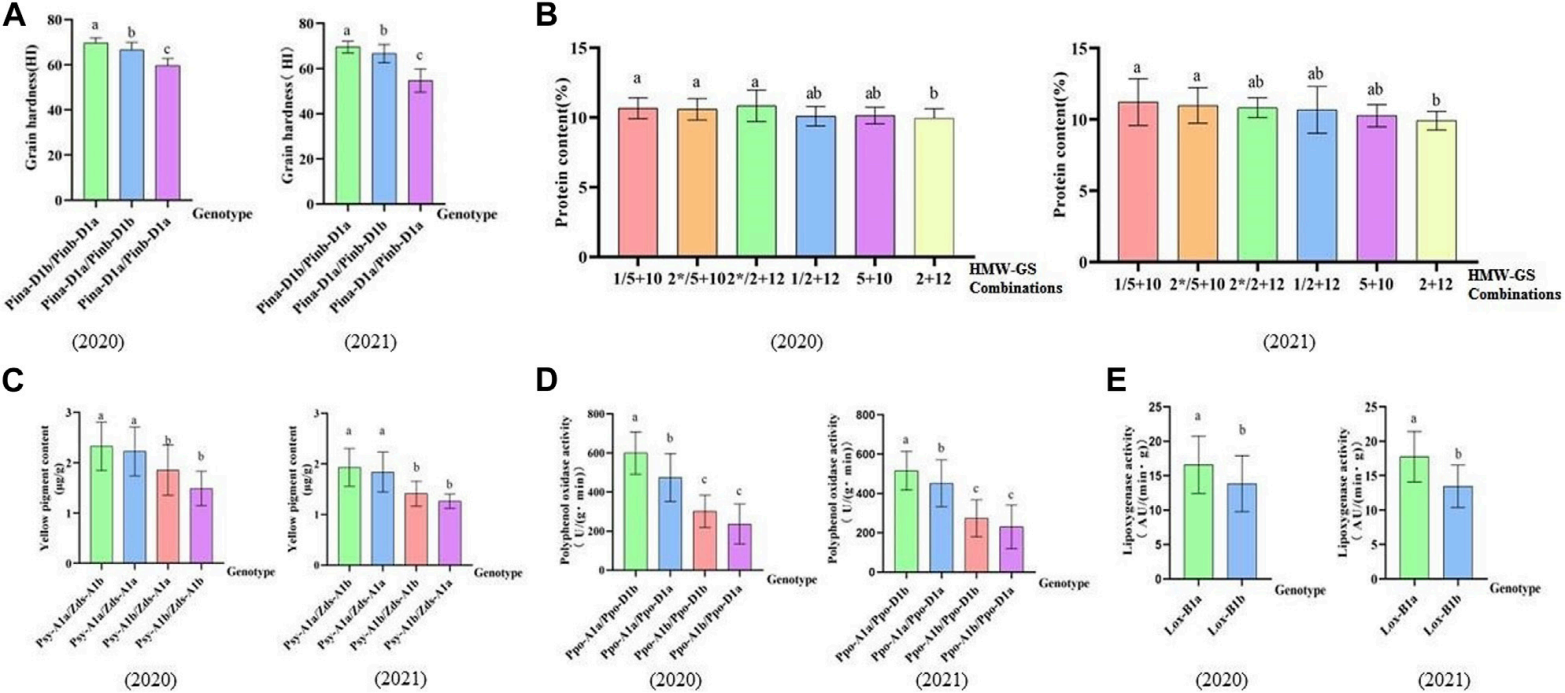
Effects of different genotypes on related quality traits. **(A–E)** Bar plots display different genotypes on quality through analyzing 2020, 2021data. **(A)** Bar plot shows the effect of different genotype combinations on grain hardness. **(B)** Bar plot indicates the influence of different HMW-GS combinations on protein content. **(C)** Bar plot demonstrates the effect of different genotypes on the yellow pigment content. **(D)** Bar plot presents the effect of different genotypes on the activity of polyphenol oxidase. **(E)** Bar plot reveals the effect of different genotypes on the activity of polyphenol oxidase.

In terms of grain hardness, we identified 46 hard wheat varieties with grain hardness higher than 70 and 30 soft wheat varieties with grain hardness less than 50, and they carry gene combinations of *Pina-D1b/Pinb-D1a* and *Pina-D1a/Pinb-D1a*, respectively. Among the 46 hard wheat varieties, 38 were from CIMMYT materials and 8 were from Chinese materials; 30 soft wheat germplasm were all Chinese materials (Figure 3A; Supplementary Tables S3, S4). As for protein content, we found five strong gluten wheat with protein content higher than 14% and 35 weak gluten wheat with protein content lower than 10%, while they carry high molecular weight Glutelin subunit combinations of 1Ax1/1Dx5+1Dy10 and 1Dx2+1Dy12, respectively. Among the 5 strong gluten wheat varieties, 4 were from CIMMYT and 1 was from China. Among the 35 weak gluten wheat varieties, 15 were from CIMMYT and 20 were from China (Figure 3B; Supplementary Table S5). In addition, we identified 3 Chinese wheat varieties with more than 3 μg/g of high yellow pigment content with genotype combinations of *Psy-A1a/Zds-A1* (Figure 3C). In terms of polyphenol oxidase, we found 21 low polyphenol oxidase activity materials with polyphenol oxidase activity lower than 200 U/(g.min), and their genotype combinations were *Ppo-A1b/Ppo-D1b* or *Ppo-A1b/Ppo-D1a* (Figure 3D). Among the 21 accessions, 15 were from CIMMYT and 6 were from China. For lipoxygenase activity, 17 low lipoxygenase active accessions with lipoxygenase activity below 10 AU/(min g) were selected, and their genotype was *Lox-B1b*. Among them, 15 were from CIMMYT and 2 were from China (Figure 3E). On the part of the 1BL/1RS translocation line, a total of 28 accessions contained this translocation and all of them were Chinse accessions. The germplasm carrying this translocation showed significantly reduced grain hardness, increased yellow pigment content and lipoxygenase activity, and reduced quality (Table 4).

**TABLE 4:** Comparison of 1BL/1RS translocation and quality traits.

Year	Phenotype	Genotype	Sample no.	Mean^a^	CV%^b^	Range
2020	grain hardness	1BL/1RS	28	63.7^B^	7.1	55.0–72.0
		non 1BL/1RS	125	67.5^A^	6.4	53.0–74.0
	protein content %	1BL/1RS	28	10.1^B^	6.0	8.5–11.4
		non 1BL/1RS	125	10.5^A^	7.8	9.0–12.6
	PPO activity AU/(min*g)	1BL/1RS	28	399.0^A^	42.6	127.0–671.0
		non 1BL/1RS	125	419.0^A^	42.6	146.7–898.0
	YP content (mg/kg)	1BL/1RS	28	2.6^A^	15.1	1.8–3.5
		non 1BL/1RS	125	2.0^B^	24.4	0.9–3.3
	LOX activity	1BL/1RS	28	16.6^A^	30.3	6.4–27.0
	(Nkat*g^-1^)	non 1BL/1RS	125	14.0^B^	27.9	3.4–25.1
2021	grain hardness	1BL/1RS	28	62.8^B^	11.1	46.5–72.4
		non 1BL/1RS	125	66.5^A^	9.4	44.5–76.6
	protein content %	1BL/1RS	28	10.5^A^	10.7	8.5–14.4
		non 1BL/1RS	125	10.6^A^	11.9	8.0–15.7
	PPO activity	1BL/1RS	28	402.4^A^	43.7	93.1–687.7
	AU/(min*g)	non 1BL/1RS	125	378.3^A^	41.6	90.4–756.9
	YP content	1BL/1RS	28	2.1^A^	19.2	1.5–2.9
	(mg/kg)	non 1BL/1RS	125	1.6^B^	23.2	0.9–2.8
	LOX activity	1BL/1RS	28	15.7^A^	26.3	9.5–25.7
	(Nkat*g^−1^)	non 1BL/1RS	125	14.1^B^	27.8	7.4–22.6

^a^ Different letters following the mean indicate significant differences based on a *t*-test (*p* < 0.01).

^b^ CV, coefficient of variation in percent.

## Discussion

### Effects of grain hardness genes on quality traits

Wheat with different grain hardness has different uses for flour. Hard wheat is suitable for making bread and noodles, and soft wheat is good for making biscuits and cakes. The hardness index (HI) values have been used to define the hard wheat (>62) and soft wheat (<46) (Sun et al., 2008). Through the measurement of grain hardness, it was found that there were 123 hard wheats in this study, accounting for 80.4% of the tested varieties. Hard wheat in both Chinese and CIMMYT materials were mainly *Pinb-D1b* mutation type. This also verified by the previous studies (Xia et al., 2005). The Chinese wheat varieties were mainly *Pina-D1a/Pinb-D1b* and *Pina-D1a/Pinb-D1a* variant types. CIMMYT wheat varieties were primarily *Pina-D1b/Pinb-D1a*, and the hardness genotype was relatively narrow. By comparing the hardness values among different variant combinations, we found that the grain hardness of wheat with *Pina-D1b* genotype was significant higher than that of wheat with *Pinb-D1a* genotype. From this, we knew the effect of *Pinb* gene on grain hardness is minor than that of *Pina* gene. The research results were consistent with previous studies (Chen et al., 2007; Yang et al., 2017; Sang et al., 2020). Grain hardness was positively correlated with crude protein content (Li et al., 2001). This research showed that the protein content of *Pina-D1b* gene was the highest, which was outstandingly higher than that of other variation combinations.

### The effect of HMW-GS on quality traits

HMW-GS accounts for 10% of the storage protein content, but it is a key factor affecting wheat processing quality (Payne et al., 1987; Zhang et al., 2009). Previous studies have proved that the *Glu-D1* site has the greatest effect, while *Glu-B1* and *Glu-A1* have less influence. The different subunits at different sites showed different effects on the processing quality of wheat flour. 1, 2*, 5 + 10 are high-quality subunits for improving the quality of gluten (Liu et al., 2005; Lu et al., 2017). Previous research showed that 2 + 12 subunits of *Glu-D1* site associated with gluten titers were the most common subunit in 123 regional varieties and modern bread wheat varieties, accounting for 63.4%, and 5 + 10 subunits associated with strong gluten bread wheat were found in 22 wheat varieties, accounting for 17.8%. Apart from these two subunits, 2.1 + 12, 2 + 12′, and 2 + 12* subunits were also found among the varieties (Sönmez et al., 2023). A previous study found *Glu-B1* and *Glu-B3* had the highest effect on the variations for gluten, dough and end-use quality traits, whereas *Glu-A1* and *Glu-D3* had the lowest impact on average. The *Glu-D1* locus had a strong impact on gluten strength but its contribution to either SDS-Sedimentation volume, gluten extensibility and bread loaf volume was minimal (Guzmán et al., 2022). However, this study revealed that there were some differences in HMW-GS composition of Chinese and CIMMYT wheat varieties. The frequencies of 1/2* and 5 + 10 subunits in CIMMYT varieties accounted for 47.1% and 42.5% of the tested varieties, respectively. For Chinese wheat varieties, the proportions of 1/2* and 5 + 10 subunits were 31.4% and 13.7%, respectively, while 1 and 5 + 10 had a positive impact on protein content (Xie et al., 2016). The protein content of combinations containing 1/2* and 5 + 10 subunits was significant higher than that of combinations with NULL and 2 + 12 subunits. Among the detected varieties, there were 11 varieties containing both high quality subunit combinations 1 + 2* and 5 + 10, all of which were CIMMYT varieties. There were 26 inferior subunit combinations of Null/2 + 12 varieties, and they all were Chinese varieties. This may also be one of the reasons for the poor quality of wheat in China.

### The influence of PPO activity, LOX activity and YP content on quality traits

PPO activity, LOX activity and YP content will affect the color of flour. High PPO activity will cause browning during flour processing and reduce the color of flour, while low PPO activity will make the flour white and bright (Fuerst et al., 2006; Leenhardt et al., 2006). In the present study, the trends of PPO alleles *PPO-A1* and *PPO-D1* in Chinese and CIMMYT wheat varieties were roughly the same, while the two major genes control PPO activity of wheat. In addition, the effect of *Ppo-A1* on PPO activity of wheat was greater than that of *Ppo-D1*, which squared with the results of previous studies (Brian and Skinner, 2011). The wide range of PPO activity between different variant combinations indicated that the selection potential was large, and low PPO activity could be selected through breeding to improve the color browning of wheat products.

Some studies suggest that carotenoid content is significantly positively correlated with flour yellowness (Hu et al., 2004). Two-year variance analysis showed that *Psy-A1* was the main gene controlling yellow pigment content. The gene combination containing *Psy-A1a* was significant higher than other combinations, as reported in the previous studies (Zhou et al., 2014).

The results showed that *Lox-B1b* and *Lox-B1a* genes accounted for 78.4% and 21.6% of the tested varieties, respectively. The activity range of 153 tested varieties in the 2 years was 3.3–27.0 Nkat*g^−1^ and 7.4–25.7 Nkat*g^−1^. The order of the difference in activity was basically the same, but the activity value of the same species changed obviously in different years. It was depend on the weather and Fusarium head blight PPO, YP, and LOX had a wide range of variations. Based on various breeding requirements, different alleles can be selected to improve the color of wheat flour.

### The effect of 1BL/1RS translocation line on quality traits

In the 1990s, the translocation line was widely used in Chinese wheat breeding because the wheat-rye 1BL/1RS translocation line had good high yield and disease resistance (Liu et al., 2020). Later studies found that wheat varieties containing 1BL/1RS had poor quality, and the utilization rate of 1BL/1RS translocated lines significantly decreased (Yu et al., 2011). A total of 27 varieties incorporated 1BL/1RS translocations, with frequencies of 32.1% in Chinese varieties and 1.4% in CIMMYT wheat varieties, respectively. The wheat varieties encompassing 1BL/1RS translocation lines had higher LOX activity, YP content and lower grain hardness. The existence of 1BL/1RS translocation line significantly affected the YP content, as previous researches mentioned (He et al., 2008). The relationship between LOX activity and the 1BL/1RS translocation line has not been studied before. In this study, the LOX of the varieties containing the 1BL/1RS translocation line and the varieties without the 1BL/1RS translocation line were quietly different. The reason may be that most varieties containing 1BL/1RS translocation line in this test also included *LOX-B1b* gene. Compared with varieties without 1BL/1RS translocation line, the protein content of varieties with 1BL/1RS translocation lines were lower in 2020, but there was no significant difference in 2021, possibly because the protein content was susceptible to the environmental impact of that year. What’s more, we found that spring wheat had high protein content. Among the 153 varieties, CIMMYT varieties were all spring wheat varieties and basically did not contain translocation line, which may be one of the reasons for the high protein content of non-1BL/1RS translocation line. Therefore, whether 1BL/1RS translocation lines have an impact on protein content still needs further study. The introduction of 1BL/1RS translocation line will lead to the deterioration of some quality traits, which is not conducive to the cultivation of high-quality wheat varieties.

## Materials and methods

### Plant varieties and field trials

A total of 153 germplasm resources were tested, including 81 from domestic varieties, and 72 from CIMMYT varieties (Supplementary Table S2). It was tested in Xiangyang Test Base for 2 years from 2019 to 2021. Each material was sown 3 g and planted in a single row with a length of 1 m, row spacing of 0.3 m. A total of 3 replicates have been used with randomized block design. The field management is the same as the local field production. After normal maturity, each material was harvested separately. When all of grains moisture were lower than 13%, phenotype of quality was initiated by using standard methods with 3 replicates per material.

### DNA extraction and identification of quality genes with molecular markers

Genomic DNA of 153 wheat varieties was isolated from their leaf using the modified cetyltrimethyl ammonium bromide (CTAB) method (Dreisigacker et al., 2016). 153 wheat varieties (Lines) were screened out by using the functional/KASP markers of quality trait genes (*PPO*, *Psy*, *Lox*, *Pina*, *Pinb*, *1BL/1RS*, *Glu-D*, and *Glu-A1*). A standard polymerase chain reaction (PCR) was performed by Dreisigacker et al. (2016). A 10 μL system was used for PCR amplification, including 1 μL DNA, 5 μL 2×Taq PCR Mix (Vazyme P112-03, China) and 1 μmol·L^−1^ primer, adding ddH_2_O to 10 μL. The PCR amplification process was as follows: predenaturation at 94°C for 5 min; denaturation at 94°C for 1 min; annealing at 50°C–66°C for 1 min; extension at 72°C for 2 min. And it was 30–35 cycles in total. Then the PCR amplification was prolonged for 10 min at 72°C and stored at 4°C. The PCR products were detected by 1.2% agarose gel electrophoresis. KASP markers were assayed by RT-PCR (CFX Connect, BIO RAD). PCR system could be summarized as follows: DNA 1 μL, KASP Master Mix (KBS-1016-001, KASP V4.0 2×Master Mix96/384, Standard Rox) 2.5 μL, primer 0.25 μL and ddH_2_O 1.25 μL, 95°C pre-denaturation for 15 min, 94°C denaturation for 15 s, annealing at 65°C for 60 s, then each cycle decreased by 0.8°C, a total of 10 cycles, finally reduced to 57°C, 94°C denaturation for 20 s, 57°C annealing for 60 s, a total of 40 cycles. The primers used were synthesized by Beijing Tsingke Biotech Co.

### Quality traits determination method

#### Grain hardness

Use wheat hardness tester JYDX100 × 40 to determine the hardness value HI of wheat samples according to the national standard GB/T21304 (Luo et al., 2018).

#### Flour protein content

Use a near-infrared instrument (NIR) 2600XT-R to determine the protein content of wheat in terms of AACC39-25.

#### LOX activity

The LOX activity was determined using the method reported by Zheng et al. (2011).

#### PPO activity

The PPO activity was determined using the method reported by Si et al. (2007).

#### YP content

AACC 14–50 has been slightly modified (Approved Methods of AACC. Method 14–50.); Pour 0.5 g of flour into a 10 mL centrifuge tube; add 2 mL of water-saturated n-butanol extract (volume ratio 5:1); stir well and shake for 30 min; centrifuge with Eppendorf at 4,000 r/min for 10 min; 200 μL supernatant is taken from pipette and poured into a 96-well microtiter plate; use a water-saturated nbutanol solution as a control; use a microplate reader to measure the absorbance of the supernatant at 436.5 nm·A, Yellow pigment content = 30.1*A (American Association of Cereal Chemists International, 2000).

### Statistical analysis

The phenotype data and genotype test results were processed and analyzed using data processing software SAS9.2 (https://www.sas.com/zh_cn/home.html) and Microsoft Excel, incorporating descriptive statistics, phenotypic correlation analysis and analysis of variance (ANOVA).

## Conclusion

Wheat is one of the most important food crops in China. Although the supply of wheat is sufficient, it still cannot meet people’s demand for good quality wheat. Therefore, screening germplasm resources to identify good-quality wheat is of great significance to improve the supply and demand structure of wheat in China. In this study, we found that there were some differences in quality of wheat varieties among Chinese and CIMMYT wheat varieties in hardness, high molecular weight wheat grain subunit protein, PPO, LOX and YP and all of them controlled by genotypes, which could be screened and introduced by marker-assisted selection. The genetic complexity of wheat quality characteristics was discovered on the basis of the detection of genotype and phenotypic traits. Numerous studies in recent years proved that molecular marker-assisted selection had sufficient accuracy in wheat quality breeding and could effectively select superior genotypes for wheat quality traits. In the future, excellent germplasm resources can be chosen by combining genotype and phenotypic traits to meet the breeding needs and speed up the process of wheat quality breeding. In this study, we identified two good strong gluten CIMMTY wheat germplasm, T. DICOCCONCI9309/AE.SQUARROSA (409)//2*PANDORA/5/WAXWING/3 /BL1496/MILAN//PI610750/4/FRNCLN/6/KACHU/BECARD//WBLL1*2/BRAMBLING and KACHU*2/3/ND643//2*PRL/2*PASTOR/4/KACHU/DANPHE as well as three higher weak gluten Chinese wheat varieties, such as EEN 1, Luo 6010 and Emai 9721. These five wheat germplasm will be used for wheat breeders to develop wheat varieties of better quality through marker-assisted selection.

## Data Availability

The datasets presented in this study can be found in online repositories. The names of the repository/repositories and accession number(s) can be found in the article/Supplementary Material.
