# Supplementary material for: Quality traits analysis of 153 wheat lines derived from CIMMYT and China
Source: Front Genet. 2023 Aug 2;14:1198835. doi: 10.3389/fgene.2023.1198835 (PMC10433775; doi:10.3389/fgene.2023.1198835)
Supplement: Supplementary file 1 [file Table1.docx]

**Table S1** Related molecular-marker and sequence information

| **Gene** | **Primer name** | **Sequence(5'-3')** | **Reference** |
| --- | --- | --- | --- |
| *Glu-D1a* | GluD1_4777-AL1 | GAAGGTGACCAAGTTCATGCTCGCTAATCCTGCGAGCAACAAAT | Rasheed et al. 2016 |
| *Glu-D1d* | GluD1_4777-AL2 | GAAGGTCGGAGTCAACGGATTGCTAATCCTGCGAGCAACAAAG |  |
|  | GluD1_4777-C | AGCCAAGGGCATGTTCTATGTCGAA |  |
| *Glu-A1a/Glu-A1b* | gluA1.1_1883_ALA | GAAGGTGACCAAGTTCATGCTAAGTGTAACTTCTCCGCAACA | Rasheed et al. 2016 |
| *Glu-A1c* | gluA1.1_1883_ALG | GAAGGTCGGAGTCAACGGATTAAGTGTAACTTCTCCGCAACG |  |
|  | gluA1.1_1873_C1 | GGCCTGGATAGTATGAAACC |  |
| *Glu-A1* | Glu-Ax1/x2*_SNP_FAM | GAAGGTGACCAAGTTCATGCTAAGTGTAACTTCTCCGCAACG | Rasheed et al. 2016 |
|  | Glu-Ax1/x2*_SNP_HEX | GAAGGTCGGAGTCAACGGATTACCTAAGTGTAACTTCTCCGCAACA |  |
|  | Glu-Ax1/x2*_SNP_C | CGAAGAAGCTTGGCCTGGATAGTAT |  |
| *Ppo-A1a* | PPO18 | AACTGCTGGCTCTTCTTCCCA |  |
| *Ppo-A1b* |  | AAGAAGTTGCCCATGTCCGC | Sun et al. 2005 |
| *Ppo-D1a* | STS01 | CGCCGACCATTTCAACAA |  |
|  |  | AGAAGGACCACAAGCCGTAG | Wang et al. 2008 |
| *Ppo-D1b* | PPO29 | TGAAGCTGCCGGTCATCTAC |  |
|  |  | AAGTTGCCCATGTCCTCGCC | He et al. 2007 |
| *Psy-A1a* | YP7A | GGACCTTGCTGATGACCGAG |  |
| *Psy-A1b* |  | TGACGGTCTGAAGTGAGAATGA | He et al. 2008 |
| *Zds-A1a* | Zds-A1-AL1 | GAAGGTGACCAAGTTCATGCTCCATGCACTTGGACCTAATAG | Rasheed et al. 2016 |
| *Zds-A1b* | Zds-A1-AL2 | GAAGGTCGGAGTCAACGGATTCCATGCACTTGGACCTAATAC |  |
|  | Zds-A1-C | AAGCCGACGCGGATTTTGAA |  |
| *Lox-B1b* | LOX18 | ACGATGTGAGTTGTGACTTGTGA |  |
|  |  | GCGCGGATAGGGGTGC | Geng et al. 2012 |
| *Pina-D1a* | Pina-D1_AL1 | GAAGGTGACCAAGTTCATGCTAACTGCCAACAACTTCGCTA |  |
| *Pina-D1b* | Pina-D1_AL2 | GAAGGTCGGAGTCAACGGATTTTGTCTAGTACCCCGCTCTG |  |
|  | Pina-D1_C | ATGAAGGCCCTCTTCCTCATAGG |  |
| *Pinb-D1* | Pinb-D1_AL1 | GAAGGTGACCAAGTTCATGCTCTCATGCTCACAGCCGCC | Rasheed et al. 2016 |
|  | Pinb-D1_AL2 | GAAGGTCGGAGTCAACGGATTCCTCATGCTCACAGCCGCT |  |
|  | Pinb-D1_C | GTCACCTGGCCCACAAAATG |  |
| *1BL/1RS* | H20 | GTTGGAAGGGAGCTCGAGCTG | Cheng et al. 2008 |
|  |  | GTTGGGCAGAAAGGTCGACATC |  |
