# Supplementary material for: Quality traits analysis of 153 wheat lines derived from CIMMYT and China
Source: Front Genet. 2023 Aug 2;14:1198835. doi: 10.3389/fgene.2023.1198835 (PMC10433775; doi:10.3389/fgene.2023.1198835)
Supplement: Supplementary file 2 [file Table2.docx]

**Table S2** List of experimental materials

| **Number** | **Cultivar(line)** | **Pedigree or Origin** |
| --- | --- | --- |
| 1 | Zhongyou 9507-1 | Zhongzuo 8131-1 derived strain 8603 |
| 2 | Xinmai 0401-2 | Zhoumai 16 / Xinmai 11 |
| 3 | Ruihua 1101-3 | N/A |
| 4 | Zhongyou 27-2 Handiduizhao | Xiaomai W029 / Yumai 28 |
| 5 | Wenmai 49-5 | N/A |
| 6 | Xinmai 23-2 | Yanzhan 4110 / Zhoumai 16 |
| 7 | Yumai 49-5 | Wen 2540 variant single plant breeding |
| 8 | Huayu 198-1 | Bainong 64 / Zhoumai 16 |
| 9 | Zhengnong 16-1 | Zgengnong 7 / Xiaoyan 6 |
| 10 | Yumai 66-5 | (MZALEONDBA-TR / Yumai 2)F6 // Lankao 90 |
| 11 | Zhoumai 22-2 | Zhoumai 12 / Wenmai 6 / Zhoumai13 |
| 12 | Zhengnong 16 | Zhengnong 7 / Xiaoyan 6 |
| 13 | Aikang 58-5 | Zhoumai 11 / Wenmai 6 / Zhengzhou 8960 |
| 14 | Zhengmai 9023-3 | {(Xiaoyan 6 / Xinong 7865) // [83(2)3-3 / 84(14)43]} F3 / 3 / Shan 213 |
| 15 | Zhoumai 22-2 | Zhoumai 12 / Wenmai 6 // Zhoumai 13 |
| 16 | Zhoumai 20 | Zhoumai 13 / Xinmai 9/ Wenmai 6 |
| 17 | Taixeu 7 | Yumai 57 / Zhoumai 16 |
| 18 | Xuke 718 | Zhoumai 13 / Luomai 4 |
| 19 | Yangao 1 | (806 / B16) // Luoyang 7602 |
| 20 | Shimai 15 | GS Jimai 38 / 92R 137 |
| 21 | Zhoumai 23 | Zhoumai 13 / Xinmai 9 |
| 22 | Zhongmai 175 | BPM27 / Jing 411 |
| 23 | Zhongmai 9 | Siyang 936 // (1523 / 1542) 83 Jian 25 |
| 24 | Zhengmai 9023 | {(Xiaoyan 6 / Xinong 65) // [83(2)3-3 / 84(14)43]}F3 / 3/ Shan 213 |
| 25 | Yangmai 12 | Yangmai 158 / 3/ TP114 / Yangmai 5 // 85-853 |
| 26 | Xumai 856 | Zhengzhou 8329 / Xuzhou 86195-14-4-4-1 |
| 27 | Gaoyou 503 | 78506 / Zaoyou 504 |
| 28 | Yannong 999 | (Yanhangxuan 2 / Lin 9511)F1 // Yan BLU14-15 |
| 29 | Yumai 47 | Yumai 2 / Baiquan 3199 |
| 30 | Tanmai 98 | Jining 13 / 942 |
| 31 | Zhongmai 895 | Zhoumai 16 / Liken 4 |
| 32 | Zhengnong 16 | Zhengnong 7 / Xiaoyan 6 |
| 33 | Taishan 21 | [(26744 / Taishan 10) F1 // Lumai 7] F4 / 3 / Lumai 18 |
| 34 | Yanzhan 1 | {[C39 / 78(6)9-2] // Jimai 5418} F3/ 3 / Yumai 18 |
| 35 | Yumai 34 (Zhengnong 7) | Aifeng 3 / Meng 201 / Niuzhute / 3 / Yumai 2 |
| 36 | Taishan 23 | 881414 / 876161 |
| 37 | Een 6 | 89-289 / 89-1012 |
| 38 | Henong 972 | N/A |
| 39 | Shannong 28 | 4142 / 6125 |
| 40 | Tanmai 98 | Jining 13 / 942 |
| 41 | Yangao 21 | (Zhoumai 16 / Yumai 49) F2 // Yumai 18 |
| 42 | Een 1 | (Luofulin 10 / 761) F1 // Sumai 3 |
| 43 | Emai 12 | Yunan 750025-12 / Emai 6 |
| 44 | Henong 326 | Aifeng 3 / Yanda 72-629 // Nongda 139 / Afulenong /// Ai |
| 45 | Heng 4338 | 4-106 / BAU 3338 |
| 46 | Jinhe 12-089 | N/A |
| 47 | Junmai K8 | Kaimai 18 / Bainong 64 |
| 48 | Nannong 9918 | Haynaldia villosa translocation line 6VS / Yangmai 158 |
| 49 | Neimai 9 | Mianyang 26 /92R1789(Tufted wheat 6VS/6AL translocation line) |
| 50 | Taishan 23 | 881414 / 876161 |
| 51 | Taishan 24 | 904017 / Zhengzhou 8329 |
| 52 | Xichang 19 | N/A |
| 53 | Xikemai 6 | Mianyang 95-325 / 92R-135 |
| 54 | Yangmai 11 | Yang 158 / 3Y. C / Jianer // Yang 85-85 |
| 55 | Yumai 34 | [Aimai 3 // (Yuxian 201 / Niuzhute)] / 3 / Yumai 2 |
| 56 | Luoyou 7 | Yutong 843 / Zhoumai 9 // Yumai 2 / Qianjinzao |
| 57 | Shimai 13 | Lin 5090 / Shi 91-5093 |
| 58 | Nongda 1193 | Shanyou 225 / J Linfen 5064 |
| 59 | Wanmai 16 | Laizhou 953 / Wen 2540 |
| 60 | Zhengmai 119 | Jimai 1 / Zhengmai 366 |
| 61 | Zhengmai 9023 | {[Xiaoyan 6 / Xinong 65) // [83(2)3-3 / 84(14)43]} F3 / Shan 213 |
| 62 | Sdau 22 | Tal (Ms2) |
| 63 | Xinyou 1 | N/A |
| 64 | Emai 195 | Shan 65 / Shanghai Baoshan 279 // Fengyou 7 |
| 65 | E 410187 | Shan 65 / Shanghai Baoshan 279 // Zhengmai 9023 |
| 66 | E 610615 | N/A |
| 67 | Zhenmai 9 | Sumai 6 / 97G59 |
| 68 | Emai 21 | 69133 / CM1168-A-ly-3y-2m-Dy |
| 69 | Emai 039 | N/A |
| 70 | Luomai 36 | N/A |
| 71 | Shannong 102 | Aimengniu II / Weimai 8 |
| 72 | Zhongmai 9 | Siyang 936 // (1523 / 1542) 83 Jian 25 |
| 73 | Zhongyi 6 | N/A |
| 74 | Luo 6010 | Yuanyang 1 / 4 / (Luo 78152 / 82C6) F1 // Mianyang 21 / 3 / Mianyang 21 |
| 75 | Emai 9721 | N/A |
| 76 | Ningmaizi 1611 | N/A |
| 77 | Luomai 163 | Luomai 6010 / Florida |
| 78 | 13212 | N/A |
| 79 | Chuanmai 65 | 98-1231 // Guinong 21 / Shenghe 3295 |
| 80 | Chuanyu 26 | Chuanyu 23 / 4 / G219 / 3 / Chuanyu 16 / H5 / Luobulin |
| 81 | Chuanmai 604 | Guinong 21 / SW3243 // Chuanmai 42 |
| 82 | CIM-1 | ROLF07*2/DIAMONDBIRD//TRCH/HUIRIVIS #1/3/BORL14 |
| 83 | CIM-2 | MEX94.27.1.20/3/SOKOLL//ATTILA/3*BCN/5/GKARON/AGSECO7846//2180/4/2*MILAN/KAUZ//PRINIA/3/BAV92 |
| 84 | CIM-3 | MUNAL#1/7/CNO79//PF70354/MUS/3/PASTOR/4/BAV92/5/FRET2/KUKUNA//FRET2/6/MILAN/KAUZ//PRINIA/3/BAV92 |
| 85 | CIM-4 | WHEAR//2*PRL/2*PASTOR/3/WAXBI/4/COPIO |
| 86 | CIM-5 | NADI/3/PBW343*2/KUKUNA*2//FRTL/PIFED/4/NADI |
| 87 | CIM-6 | ATTILA/3*BCN//BAV92/3/TILHI/4/SUP152/5/SUP152/6/KACHU#1/KIRITATI//KACHU |
| 88 | CIM-7 | MUNAL*2//WAXWING*2/TUKURU/3/MUCUY |
| 89 | CIM-8 | FITIS/3/KACHU #1/KIRITATI//KACHU |
| 90 | CIM-9 | BORL14*2//MUNAL #1/FRANCOLIN #1 |
| 91 | CIM-10 | MUU/5/WBLL1*2/4/YACO/PBW65/3/KAUZ*2/TRAP//KAUZ/6/WBLL1*2/SHAMA*2/7/PRL/2*PASTOR*2//FH6-1-7 |
| 92 | CIM-11 | KIRITATI//PRL/2*PASTOR/5/OASIS/SKAUZ//4*BCN/3/PASTOR/4/KAUZ*2/YACO//KAUZ/6/KIRITATI//PRL/2*PASTOR/7/KSW/SAUAL//SAUAL/8/KIRITATI//PRL/2*PASTOR/5/OASIS/SKAUZ//4*BCN/3/PASTOR/4/KAUZ*2/YACO//KAUZ/6/KIRITATI//PRL/2*PASTOR |
| 93 | CIM-12 | BABAX/LR42//BABAX*2/3/KUKUNA/4/CROSBILL#1/5/BECARD*2/6/KACHU/KINDE |
| 94 | CIM-13 | BORL14/CHIPAK |
| 95 | CIM-14 | CHIBIA//PRLII/CM65531/3/MISR2*2/4/HUW234+LR34/PRINIA//PBW343*2/KUKUNA/3/ROLF07 |
| 96 | CIM-15 | NELOKI//SOKOLL/EXCALIBUR |
| 97 | CIM-16 | NELOKI//SOKOLL/EXCALIBUR |
| 98 | CIM-17 | KASUKO |
| 99 | CIM-18 | NELOKI//SOKOLL/EXCALIBUR |
| 100 | CIM-19 | WBLL1*2/BRAMBLING//WBLL1*2/BRAMBLING/3/2*BORL14 |
| 101 | CIM-20 | CIRO16/2*BORL14 |
| 102 | CIM-21 | WBLL1*2/CHAPIO//HEILO/3/2*KSW/SAUAL//SAUAL |
| 103 | CIM-22 | VILLAJUAREZF2009/SOLALA//WBLL1*2/BRAMBLING/3/PBW343*2/KUKUNA*2//FRTL/PIFED |
| 104 | CIM-23 | KOKILA/BOKOTA |
| 105 | CIM-24 | VILLAJUAREZF2009/3/T.DICOCCONPI94625/AE.SQUARROSA(372)//3*PASTOR/4/WBLL1*2/BRAMBLING/5/VALI/6/SUP152//WBLL1*2/BRAMBLING |
| 106 | CIM-25 | ZINCOL/8/FRANCOLIN #1/7/REH/HARE//2*BCN/3/CROC_1/AE.SQUARROSA (213)//PGO/4/HUITES/5/T.SPELTAPI348599/6/REH/HARE//2*BCN/3/CROC_1/AE.SQUARROSA (213)//PGO/4/HUITES |
| 107 | CIM-26 | WHEAR/KUKUNA/3/C80.1/3*BATAVIA//2*WBLL1/4/T.DICOCCONPI94625/AE.SQUARROSA(372)//SHA4/CHIL/5/WHEAR/KUKUNA/3/C80.1/3*BATAVIA//2*WBLL1/6/VILLAJUAREZF2009/3/T.DICOCCONPI94625/AE.SQUARROSA(372)//3*PASTOR/4/WBLL1*2/BRAMBLING/7/TRAP#1/BOW/3/VEE/PJN//2* |
| 108 | CIM-27 | DANPHE#1*2/3/T.DICOCCONPI94625/AE.SQUARROSA(372)//SHA4/CHIL/4/WBLL1*2/KURUKU//HEILO/5/WBLL1*2/KURUKU//HEILO |
| 109 | CIM-28 | KVZ/PPR47.89C//TACUPETOF2001*2/BRAMBLING/3/2*TACUPETOF2001*2/BRAMBLING/4/KACHU/5/KACHU#1/3/C80.1/3*BATAVIA//2*WBLL1/4/KACHU |
| 110 | CIM-29 | T.DICOCCONCI9309/AE.SQUARROSA(409)//2*PANDORA/5/WAXWING/3/BL1496/MILAN//PI610750/4/FRNCLN/6/KACHU/BECARD//WBLL1*2/BRAMBLING |
| 111 | CIM-30 | SHAKTI/2*BORL14 |
| 112 | CIM-31 | BORL14/FITIS |
| 113 | CIM-32 | BORL14/FITIS |
| 114 | CIM-33 | UP2338*2/KKTS*2//YANAC/3/WAXBI |
| 115 | CIM-34 | BOKOTA//KFA/2*KACHU |
| 116 | CIM-35 | VORB/FISCAL//WBLL1*2/KURUKU/3/QUAIU/4/KACHU/KINDE |
| 117 | CIM-36 | PICUS/3/KAUZ*2/BOW//KAUZ/4/KKTS/5/T.SPELTAPI348530/6/2*FRANCOLIN #1/7/KACHU/KIRITATI |
| 118 | CIM-37 | KACHU*2/SUP152 |
| 119 | CIM-38 | KACHU*2/SUP152 |
| 120 | CIM-39 | SAUAL/MUTUS//2*KACHU/KIRITATI |
| 121 | CIM-40 | TACUPETOF2001*2/BRAMBLING//WBLL1*2/BRAMBLING/6/WBLL1*2/KURUKU*2/5/REH/HARE//2*BCN/3/CROC_1/AE.SQUARROSA(213)//PGO/4/HUITES/7/BAV92//IRENA/KAUZ/3/HUITES/4/2*ROLF07 |
| 122 | CIM-41 | BOKOTA//BECARD/QUAIU #1/3/BOKOTA |
| 123 | CIM-42 | FRET2*2/SHAMA*2/4/BOW/URES//2*WEAVER/3/CROC_1/AE.SQUARROSA (213)//PGO/5/KFA/2*KACHU/6/FRET2*2/SHAMA//KACHU |
| 124 | CIM-43 | ATTILA*2/PBW65*2//TNMU*2/3/KFA/2*KACHU |
| 125 | CIM-44 | BORL14*2//KFA/2*KACHU |
| 126 | CIM-45 | BORL14*2//KFA/2*KACHU |
| 127 | CIM-46 | WBLL1/FRET2//PASTOR*2/3/MURGA/6/KSW/5/2*ALTAR84/AE.SQUARROSA(221)//3*BORL95/3/URES/JUN//KAUZ/4/WBLL1/7/KFA/2*KACHU |
| 128 | CIM-47 | BAJ #1/3/KIRITATI//ATTILA*2/PASTOR*2/4/MUTUS*2/TECUE #1 |
| 129 | CIM-48 | MUTUS/AKURI//SUP152/BAJ #1 |
| 130 | CIM-49 | BAJ #1/AKURI//KACHU/KIRITATI |
| 131 | CIM-50 | BORL14*2//KFA/2*KACHU |
| 132 | CIM-51 | BORL14*2//BECARD/QUAIU #1 |
| 133 | CIM-52 | SAUAL/YANAC//SAUAL/3/2*KUTZ |
| 134 | CIM-53 | KUTZ*2//KFA/2*KACHU |
| 135 | CIM-54 | BECARD#1/5/KIRITATI/4/2*SERI.1B*2/3/KAUZ*2/BOW//KAUZ*2/6/KFA/2*KACHU |
| 136 | CIM-55 | KUTZ//KACHU/DANPHE |
| 137 | CIM-56 | MUCUY//KACHU/KIRITATI |
| 138 | CIM-57 | KACHU#1/3/T.DICOCCONPI94624/AE.SQUARROSA(409)//BCN/4/2*KACHU/5/SWSR22T.B./KACHU//2*KACHU |
| 139 | CIM-58 | CHEWINK #1/FRNCLN/3/WBLL1*2/BRAMBLING*2//BAVIS |
| 140 | CIM-59 | BAJ #1/TECUE #1//MUTUS*2/TECUE #1/3/MUCUY |
| 141 | CIM-60 | KACHU#1//WBLL1*2/KUKUNA*2/6/BECARD#1/5/KIRITATI/4/2*SERI.1B*2/3/KAUZ*2/BOW//KAUZ |
| 142 | CIM-61 | MUTUS*2/MUU//2*MUCUY |
| 143 | CIM-62 | MUTUS*2/MUU//2*MUCUY |
| 144 | CIM-63 | MUNAL/WESTONIA//2*BORL14 |
| 145 | CIM-64 | FRET2/TUKURU//FRET2/3/MUNAL #1*2/4/KACHU/KIRITATI |
| 146 | CIM-65 | BAJ #1*2/HUIRIVIS #1*2//TAITA |
| 147 | CIM-66 | FRNCLN*2/BECARD//2*BORL14 |
| 148 | CIM-67 | KASUKO |
| 149 | CIM-68 | MUTUS//WBLL1*2/BRAMBLING/3/WBLL1*2/BRAMBLING/4/KFA/2*KACHU |
| 150 | CIM-69 | KENYA SUNBIRD/KACHU//KACHU/KIRITATI |
| 151 | CIM-70 | KACHU*2/3/ND643//2*PRL/2*PASTOR/4/KACHU/DANPHE |
| 152 | CIM-71 | MANKU/6/WHEAR/KUKUNA/3/C80.1/3*BATAVIA//2*WBLL1/5/PRL/2*PASTOR/4/CHOIX/STAR/3/HE1/3*CNO79//2*SERI |
| 153 | CIM-72 | WHEAR/KUKUNA/3/C80.1/3*BATAVIA//2*WBLL1/4/T.DICOCCON PI94625/AE.SQUARROSA (372)//3*PASTOR/5/WHEAR/KUKUNA/3/C80.1/3*BATAVIA//2*WBLL1/6/2*MANKU |

1 N/A, Not Applicable.
