# Supplementary material for: Quality traits analysis of 153 wheat lines derived from CIMMYT and China
Source: Front Genet. 2023 Aug 2;14:1198835. doi: 10.3389/fgene.2023.1198835 (PMC10433775; doi:10.3389/fgene.2023.1198835)
Supplement: Supplementary file 3 [file DataSheet1.pdf]

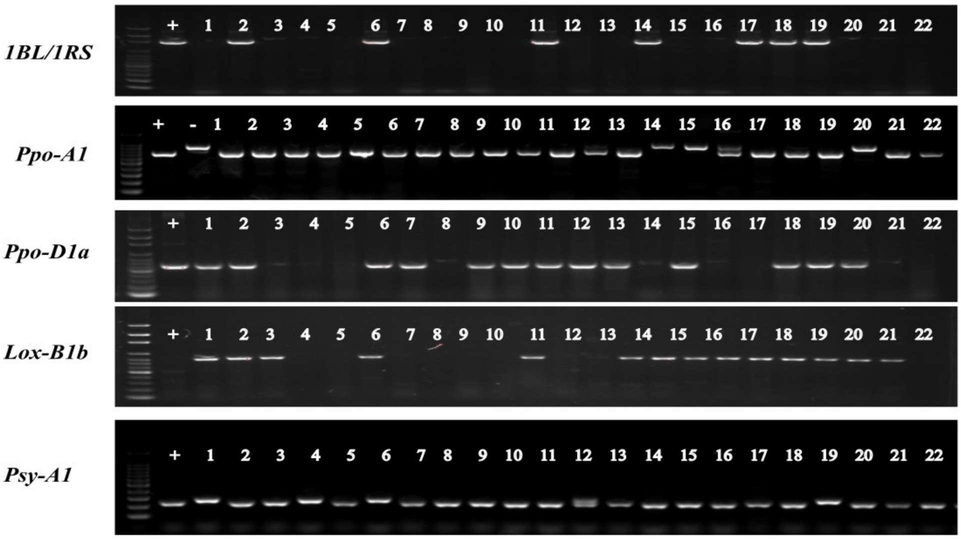

(a)

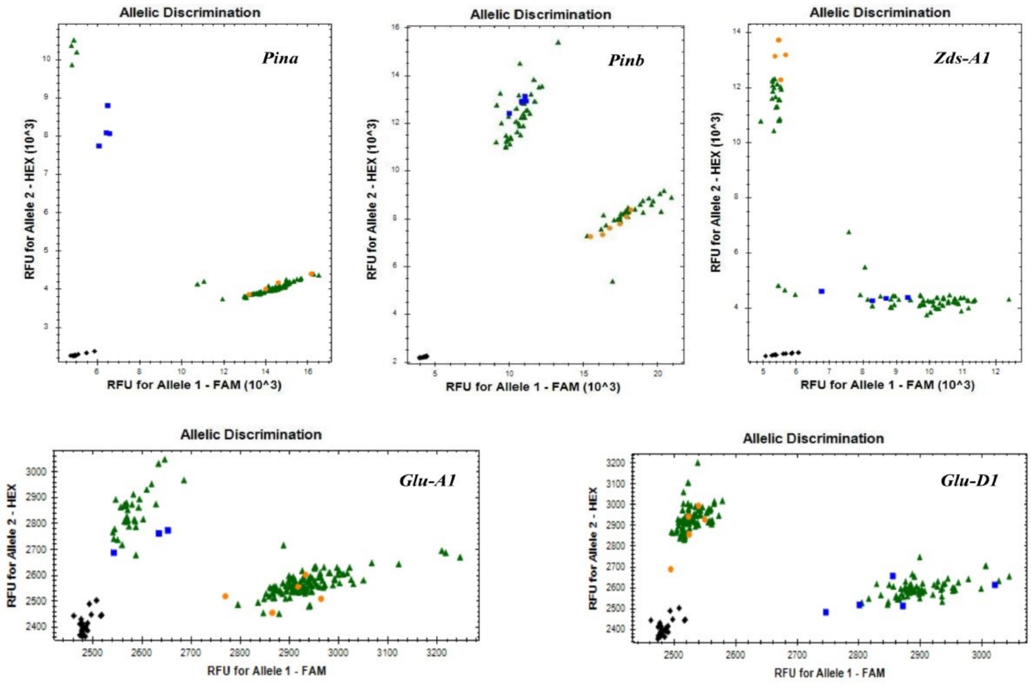

(b)

Figure 1. Part of PCR amplification of wheat cultivars (a)**.** The detection results of partial materials with quality genes (a) Common PCR results. +: Positive control; -: Negative control; 1-22: Test materials; (b) Kasp detection results of *Glu-A1* and *Glu-D1*. Black: ddH_2O; Blue and orange: control varieties; Green: Test Material

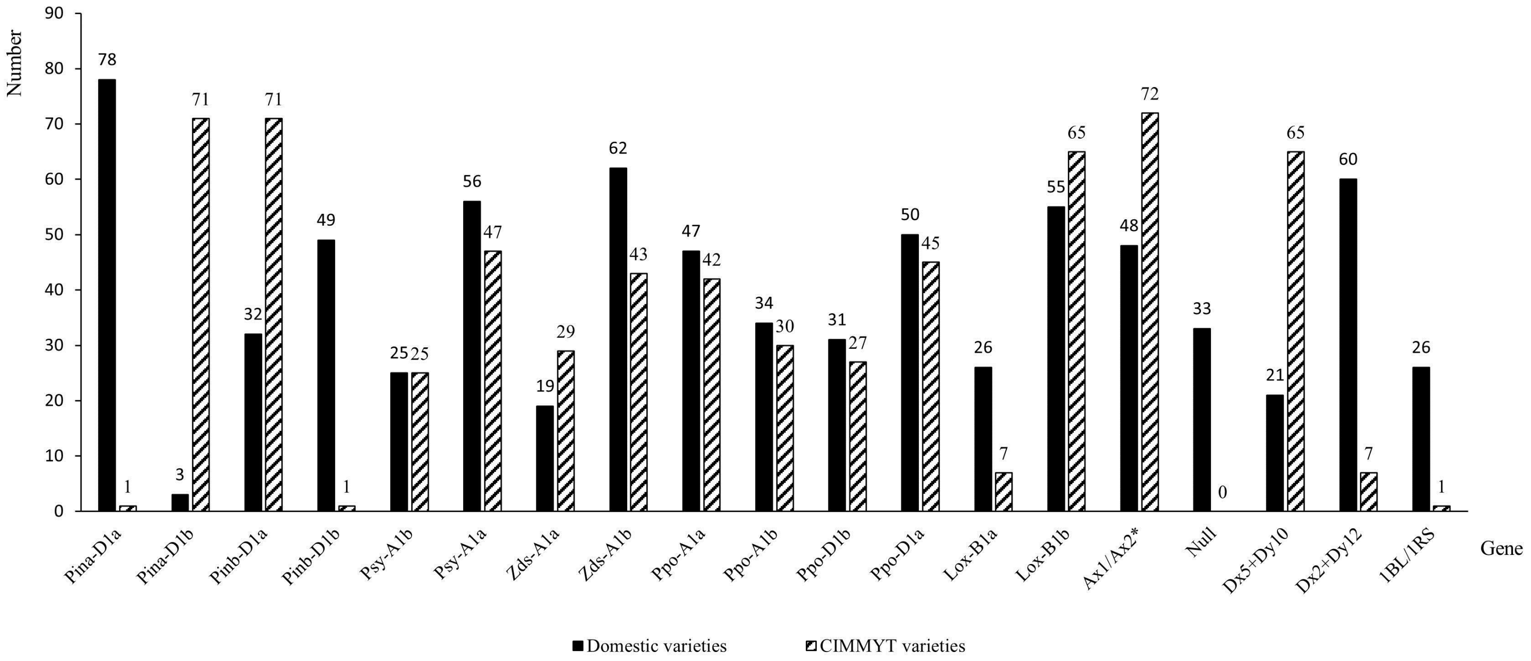

Number
90
80
70
60
50
40
30
20
10
0
78
1
3
71
32
71
49
1
25
25
56
47
19
29
62
43
47
42
34
30
31
27
50
45
26
7
55
65
48
72
33
0
21
65
60
7
26
1
Pina-D1a
Pina-D1b
Pinb-D1a
Pinb-D1b
Psy-A1b
Psy-A1a
Zds-A1a
Zds-A1b
Ppo-A1a
Ppo-A1b
Ppo-D1b
Ppo-D1a
Lox-B1a
Lox-B1b
Ax1/Ax2*
Null
Dx5+Dy10
Dx2+Dy12
1BL/1RS
Gene
Domestic varieties
CIMMYT varieties

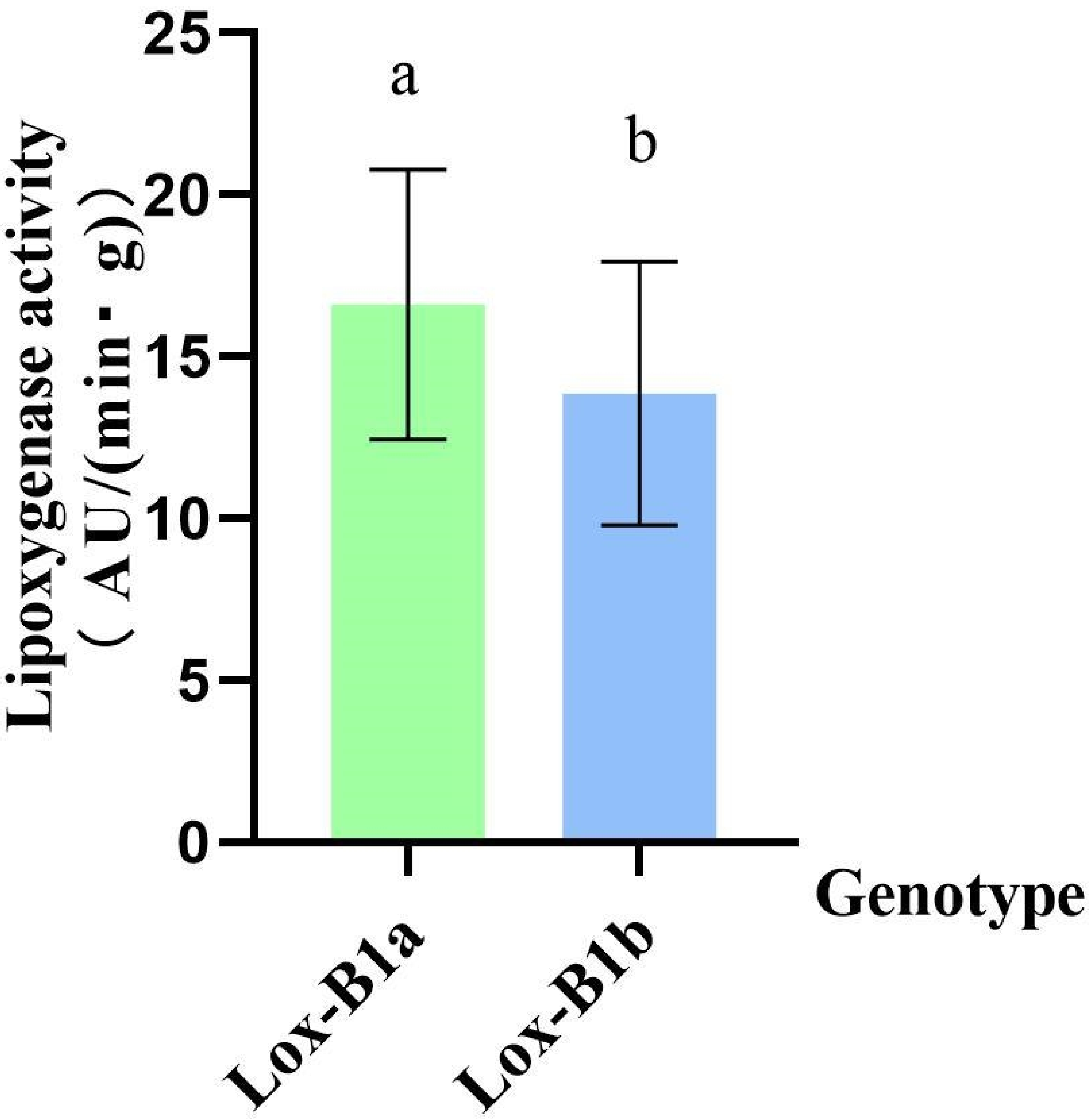

Lipoxygenase activity
(AU/(min · g))
25
20
15
10
5
0
a
b
Genotype
Lox-B1a
Lox-B1b

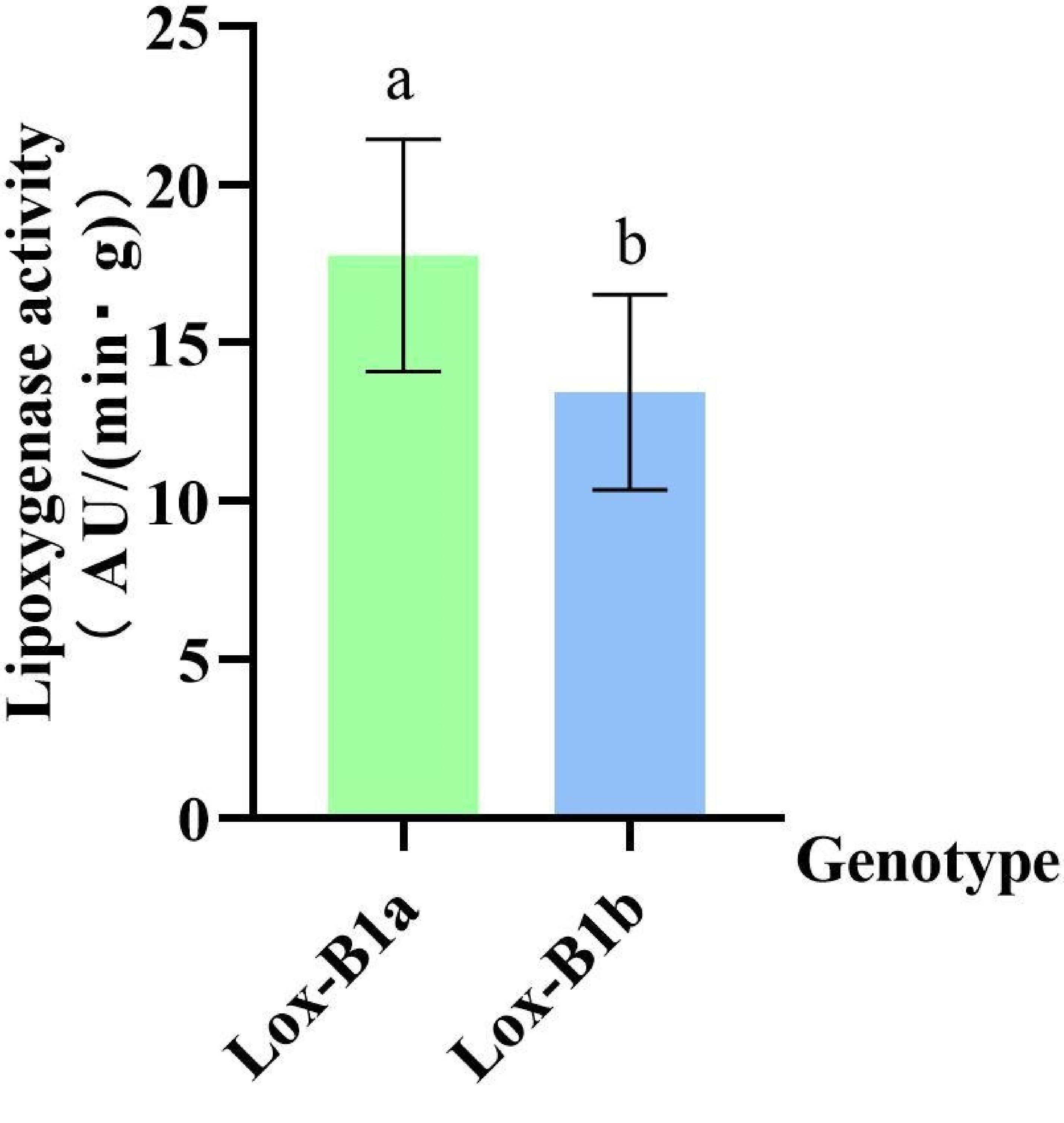

Lipoxygenase activity
（AU/(min · g)）
25
20
15
10
5
0
a
b
Genotype
Lox-B1a
Lox-B1b

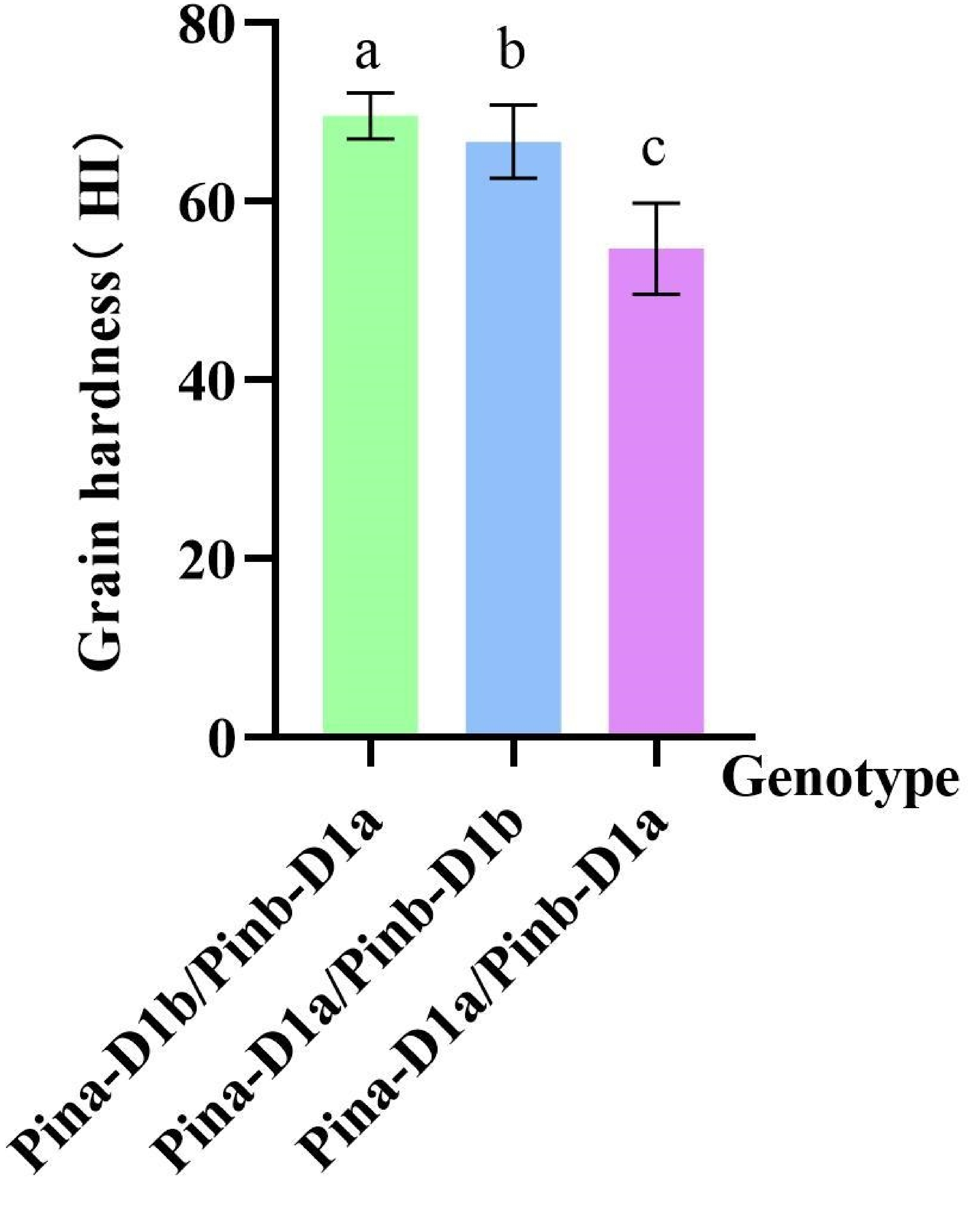
80
60
40
20
0
Grain hardness（HI）
a
b
c
Genotype
Pina-D1b/Pinb-D1a
Pina-D1a/Pinb-D1b
Pina-D1a/Pinb-D1a

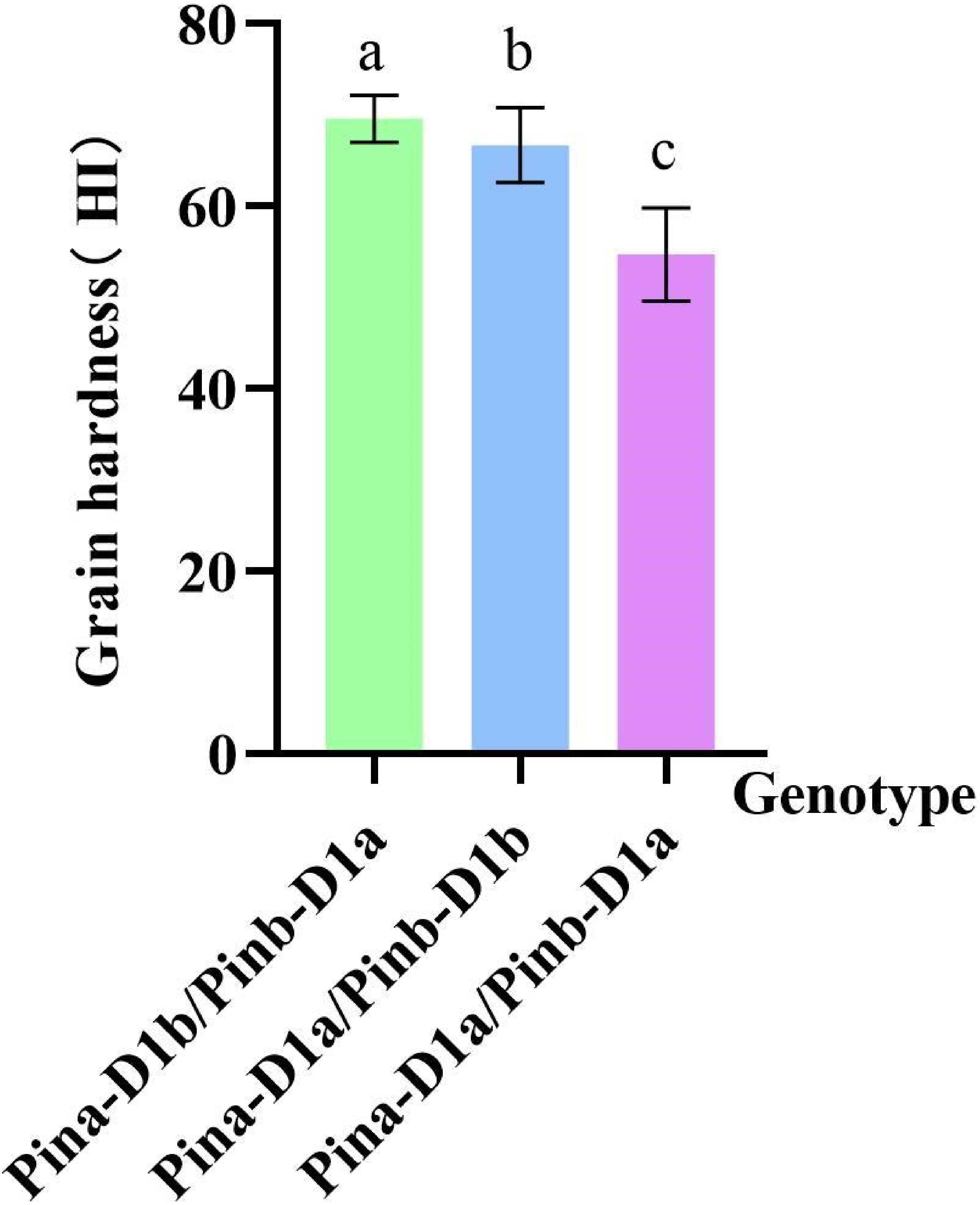

Grain hardness（HI）
80
60
40
20
0
a
b
c
Genotype
Pina-D1b/Pinb-D1a
Pina-D1a/Pinb-D1b
Pina-D1a/Pinb-D1a

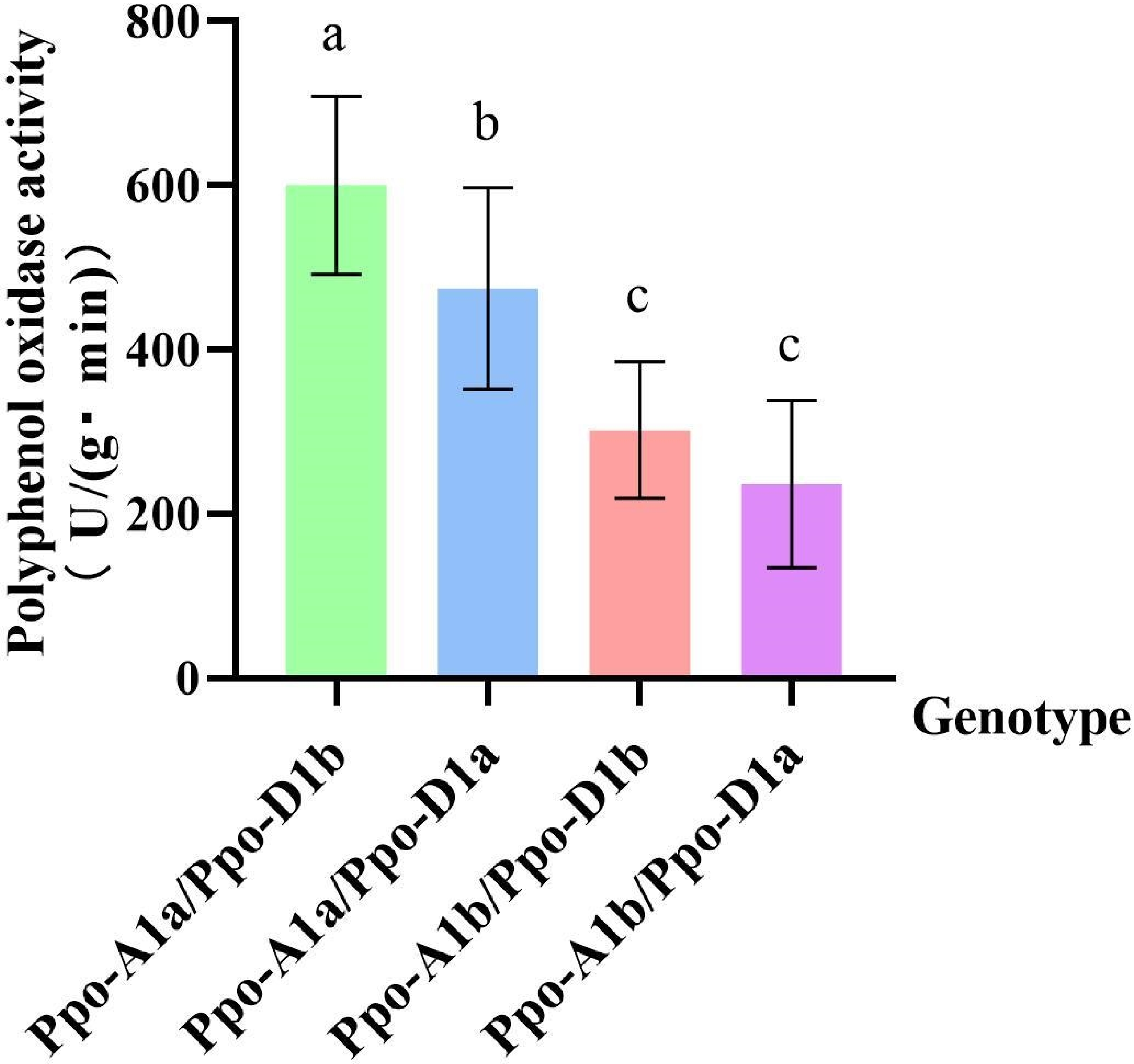

Polyphenol oxidase activity
(U/(g· min))
800
600
400
200
0
a
b
c
c
Genotype
Ppo-A1a/Ppo-D1b
Ppo-A1a/Ppo-D1a
Ppo-A1b/Ppo-D1b
Ppo-A1b/Ppo-D1a

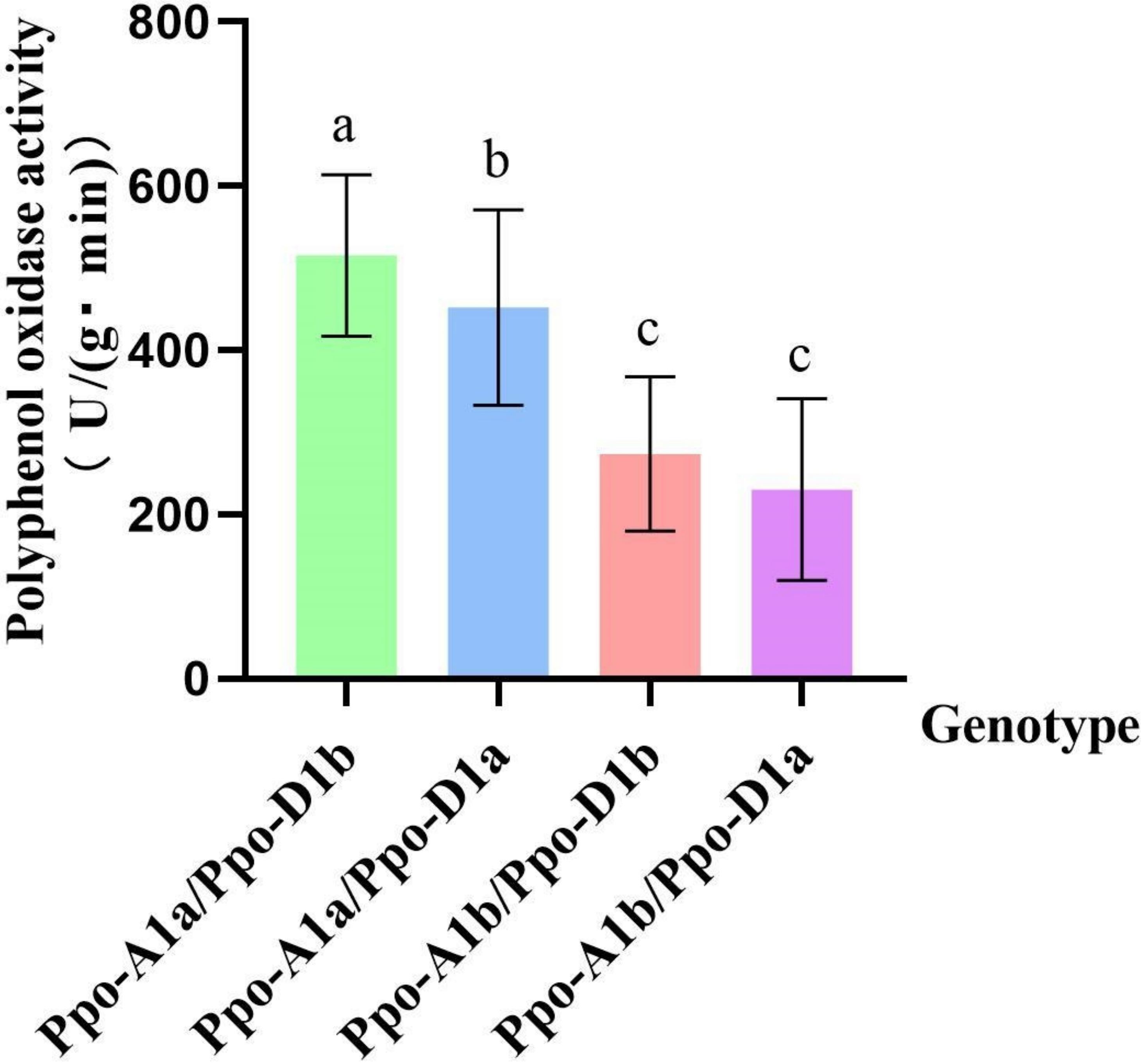

Polyphenol oxidase activity
(U/(g · min))
800
600
400
200
0
a
b
c
c
Genotype
Ppo-A1a/Ppo-D1b
Ppo-A1a/Ppo-D1a
Ppo-A1b/Ppo-D1b
Ppo-A1b/Ppo-D1a

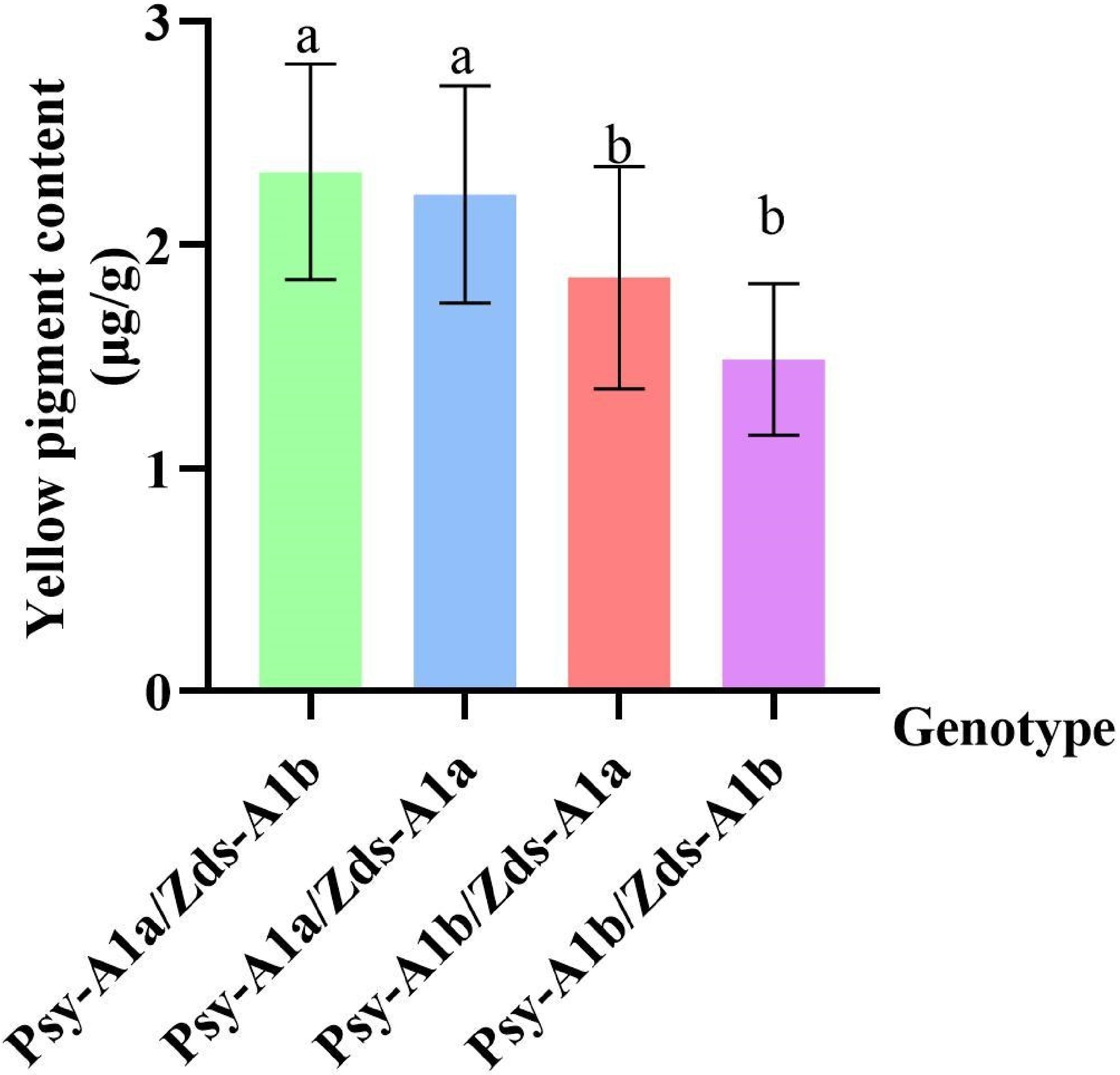

Yellow pigment content (μg/g)
3
2
1
0
a
a
b
b
Genotype
Psy-A1a/Zds-A1b
Psy-A1a/Zds-A1a
Psy-A1b/Zds-A1a
Psy-A1b/Zds-A1b

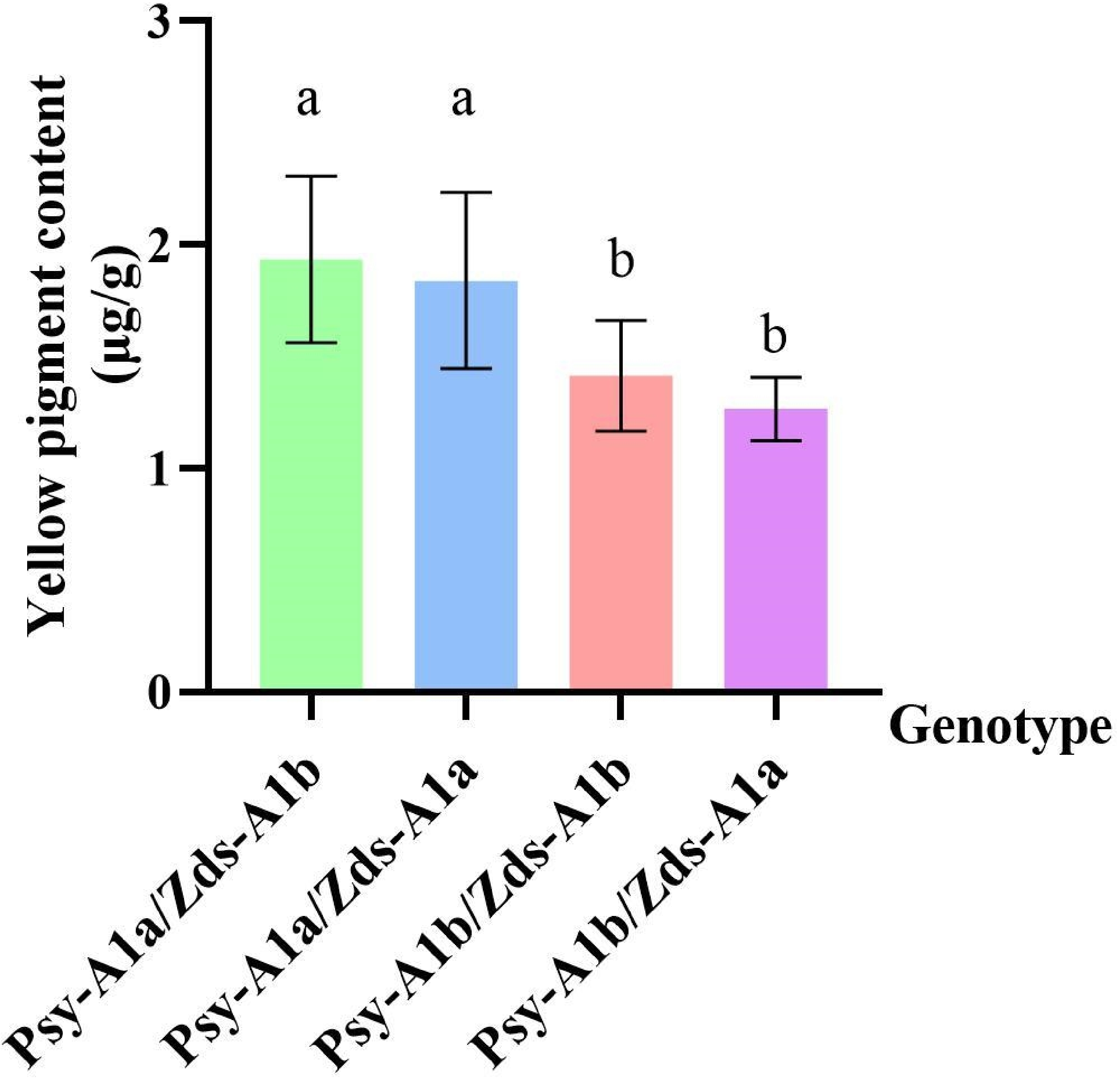

Yellow pigment content (µg/g)
3
2
1
0
a
a
b
b
Genotype
Psy-A1a/Zds-A1b
Psy-A1a/Zds-A1a
Psy-A1b/Zds-A1b
Psy-A1b/Zds-A1a

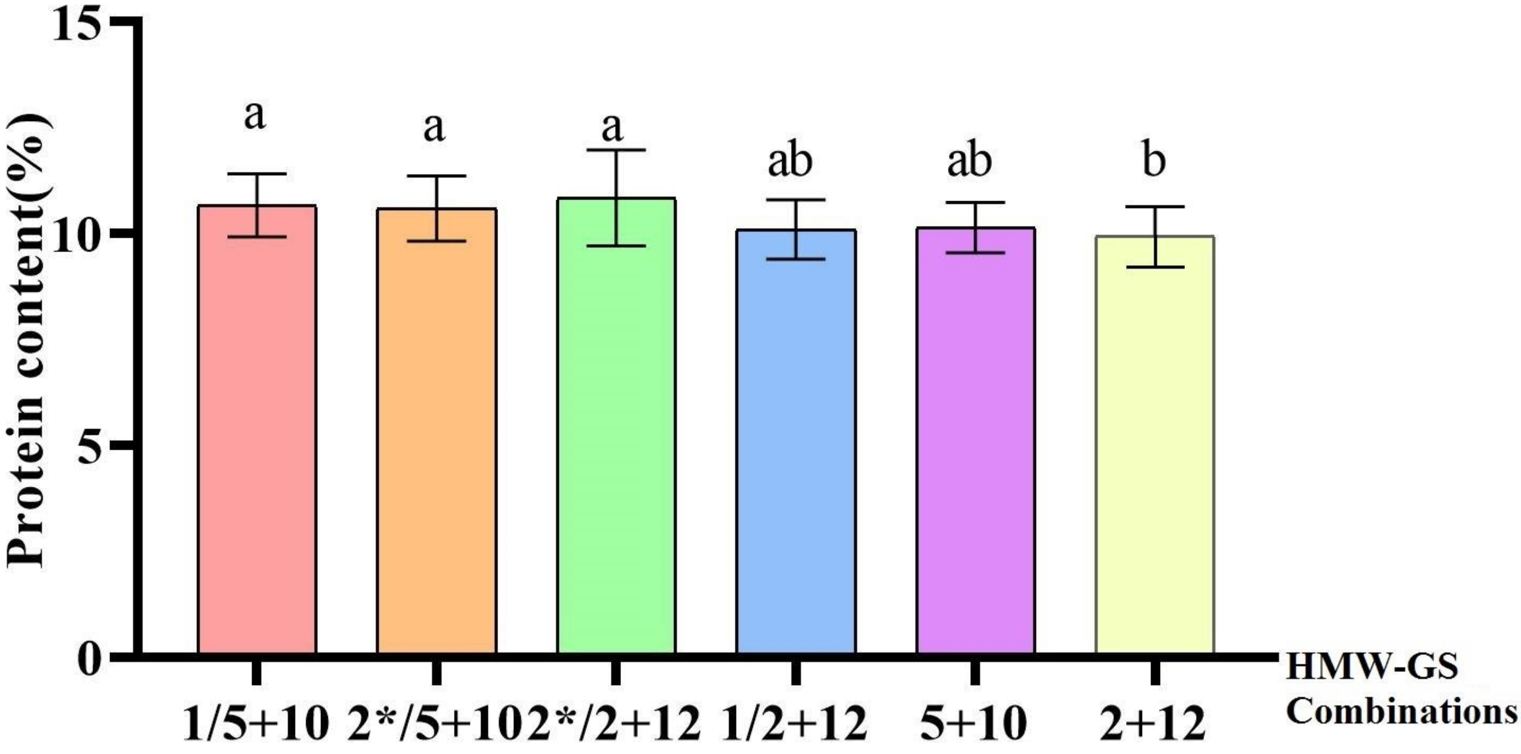

15
10
5
0
Protein content(%)
a
a
a
ab
ab
b
1/5+10
2*/5+10
2*/2+12
1/2+12
5+10
2+12
HMW-GS
Combinations

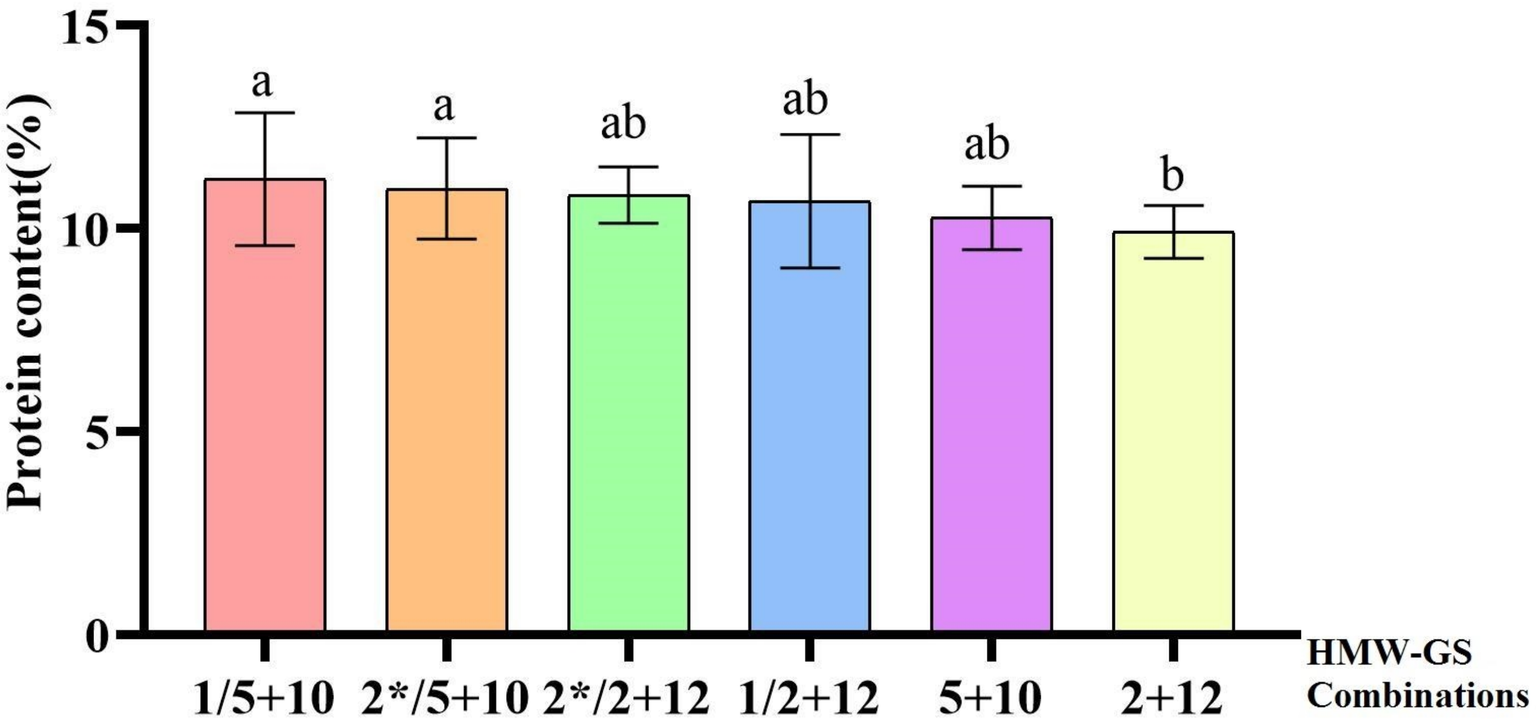

Protein content(%)
15
10
5
0
a
a
ab
ab
ab
b
1/5+10
2*/5+10
2*/2+12
1/2+12
5+10
2+12
HMW-GS
Combinations

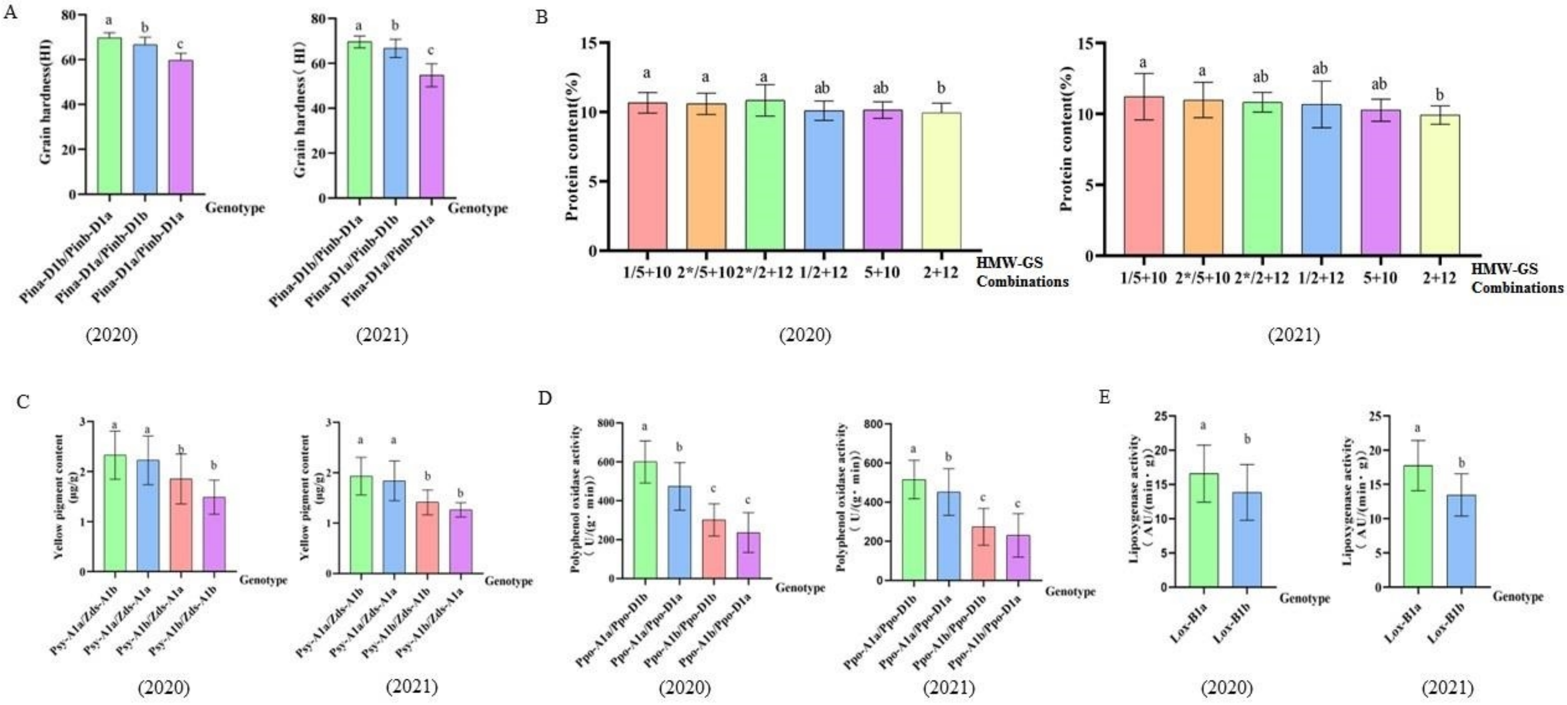
A
Grain hardness(HI)
Grain hardness(HI)
Genotype
Pina-D1b/Pinb-D1a
Pina-D1a/Pinb-D1b
Pina-D1a/Pinb-D1a
(2020)
(2021)
B
Protein content(%)
HMW-GS Combinations
1/5+10
2*/5+10
2*/2+12
1/2+12
5+10
2+12
(2020)
(2021)
C
Yellow pigment content (μg/g)
Genotype
Psy-A1a/Zds-A1b
Psy-A1a/Zds-A1a
Psy-A1b/Zds-A1a
Psy-A1b/Zds-A1b
(2020)
(2021)
D
Polyphenol oxidase activity (U/(g · min))
Genotype
Ppo-A1a/Ppo-D1b
Ppo-A1a/Ppo-D1a
Ppo-A1b/Ppo-D1b
Ppo-A1b/Ppo-D1a
(2020)
(2021)
E
Lipoxygenase activity (AU/(min · g))
Genotype
Lox-B1a
Lox-B1b
(2020)
(2021)
