# Supplementary material for: Quality traits analysis of 153 wheat lines derived from CIMMYT and China
Source: Front Genet. 2023 Aug 2;14:1198835. doi: 10.3389/fgene.2023.1198835 (PMC10433775; doi:10.3389/fgene.2023.1198835)
Supplement: Supplementary file 4 [file Table5.docx]

**Table S5** Comparison the protein content among different HMW-GS combinations

| **Year** | **subunits** | **domestic varieties Number** | **CIMMYT varieties**  **Number** | **Mean^1^** | **SD^2^** | **CV(%)^3^** | **Range** |
| --- | --- | --- | --- | --- | --- | --- | --- |
| 2020 | 1/2 /5+10 | 14 | 65 | 10.6^A^ | 0.76 | 7.1 | 9.2-12.6 |
|  | 1/2 /2+12 | 34 | 7 | 10.2^AB^ | 0.84 | 8.2 | 8.9-12.5 |
|  | Null /5+10 | 7 | 0 | 10.1^AB^ | 0.59 | 5.8 | 9.7-11.1 |
|  | Null /2+12 | 26 | 0 | 9.9^B^ | 0.68 | 6.8 | 8.5-11.1 |
| 2021 | 1/2/ 5+10 | 14 | 65 | 10.8^A^ | 1.28 | 11.9 | 8.2-15.7 |
|  | 1/2 /2+12 | 34 | 7 | 10.6^AB^ | 1.35 | 12.8 | 8.0-14.4 |
|  | Null/5+10 | 7 | 0 | 10.3^AB^ | 0.78 | 7.6 | 9.4-11.6 |
|  | Null/2+12 | 26 | 0 | 9.9^B^ | 0.65 | 6.5 | 8.7-10.8 |

1 Different letters following the mean indicate significant differences based on a *t* test (*P* < 0.01)

2 SD, standard deviation.

3 CV, coefficient of variation in percent
