# Supplementary material for: Quality traits analysis of 153 wheat lines derived from CIMMYT and China
Source: Front Genet. 2023 Aug 2;14:1198835. doi: 10.3389/fgene.2023.1198835 (PMC10433775; doi:10.3389/fgene.2023.1198835)
Supplement: Supplementary file 5 [file Table3.docx]

**Table S3** Comparison the grain hardness among different genotypes

| **Year** | **Genotype** | **domestic varieties Number** | **CIMMYT varieties**  **Number** | **Mean^1^** | **SD^2^** | **CV(%)^3^** | **Range** |
| --- | --- | --- | --- | --- | --- | --- | --- |
| 2020 | *Pina-D1a/Pinb-D1a* | 29 | 0 | 59.8 ^C^ | 3.07 | 5.1 | 55.0-67.0 |
|  | *Pina-D1b/Pinb-D1a* | 3 | 71 | 69.7^A^ | 2.23 | 3.2 | 63.0-74.0 |
|  | *Pina-D1a/Pinb-D1b* | 49 | 1 | 66.7^B^ | 3.18 | 4.8 | 57.0-72.0 |
| 2021 | *Pina-D1a/Pinb-D1a* | 29 | 0 | 54.8^C^ | 5.00 | 9.1 | 47.5-67.8 |
|  | *Pina-D1b/Pinb-D1a* | 3 | 71 | 69.5^A^ | 2.56 | 3.7 | 63.2-76.6 |
|  | *Pina-D1a/Pinb-D1b* | 49 | 1 | 66.8^B^ | 3.64 | 5.5 | 54.5-72.6 |

1 Different letters following the mean indicate significant differences based on a *t* test (*P* < 0.01).

2 SD, standard deviation.

3 CV, coefficient of variation in percent.
