# Supplementary material for: Quality traits analysis of 153 wheat lines derived from CIMMYT and China
Source: Front Genet. 2023 Aug 2;14:1198835. doi: 10.3389/fgene.2023.1198835 (PMC10433775; doi:10.3389/fgene.2023.1198835)
Supplement: Supplementary file 6 [file Table4.docx]

**Table S4** Comparison the protein content among different genotypes

| **Year** | **Genotype** | **domestic varieties Number** | **CIMMYT varieties**  **Number** | **Mean^1^** | **SD^2^** | **CV(%)^3^** | **Range** |
| --- | --- | --- | --- | --- | --- | --- | --- |
| 2020 | *Pina-D1a/Pinb-D1a* | 29 | 0 | 9.8 ^C^ | 0.54 | 5.5 | 8.5-11.0 |
|  | *Pina-D1b/Pinb-D1a* | 3 | 71 | 10.7^A^ | 0.80 | 7.4 | 9.2-12.6 |
|  | *Pina-D1a/Pinb-D1b* | 49 | 1 | 10.3^B^ | 0.72 | 7.0 | 8.9-11.7 |
| 2021 | *Pina-D1a/Pinb-D1a* | 29 | 0 | 9.9^B^ | 1.09 | 10.9 | 8.0-13.1 |
|  | *Pina-D1b/Pinb-D1a* | 3 | 71 | 10.9^A^ | 1.34 | 12.4 | 8.2-15.7 |
|  | *Pina-D1a/Pinb-D1b* | 49 | 1 | 10.5^AB^ | 1.02 | 9.7 | 8.5-14.4 |

1 Different letters following the mean indicate significant differences based on a *t* test (*P* < 0.01)

2 SD, standard deviation.

3 CV, coefficient of variation in percent.
